# Shifts in phytoplankton communities in response to water parameters and large branchiopod filter feeders in kettle hole ponds of farmland landscape

**DOI:** 10.1038/s41598-025-01060-9

**Published:** 2025-05-21

**Authors:** Sofia Celewicz, Bartłomiej Gołdyn

**Affiliations:** 1 Department of Botany, Faculty of Agriculture, Horticulture and Biotechnology, Poznań University of Life Sciences, Dąbrowskiego 159, 60-594 Poznań, Poland; 2 Department of General Zoology, Faculty of Biology, Adam Mickiewicz University in Poznań, Uniwersytetu Poznańskiego 6, 61-614 Poznań, Poland

**Keywords:** Microalgae, Temporary ponds, Euglenoids, Astatic water bodies, Macroinvertebrates, Chlorophytes, Physical and chemical parameters, Ecology, Plant sciences, Zoology, Environmental sciences

## Abstract

Temporary ponds in agricultural landscapes, highly vulnerable to anthropogenic pressure and climatic variation, constitute unique habitats for microalgae and endangered filter feeders (large branchiopods). Such ponds play a crucial role in the functioning of farmland ecosystem, yet they remain largely understudied. Our study is the first to examine changes in phytoplankton communities in temporary kettle hole ponds in relation to rapid shifts in biotic (large branchiopods) and physical and chemical parameters of water. We conducted our research over a three-year cycle (in nine ponds in western Poland), with sampling starting in spring when inundation usually occurs, and continuing until the ponds dried out. Among 406 phytoplankton taxa (mainly euglenoids, but also diatoms and chlorophytes), locally rare species (e.g. *Desmatractum indutum*) were noted. When branchiopods occurred in the ponds, the phytoplankton communities shifted towards unicellular chlorophytes. Furthermore, nitrogen forms, temperature, conductivity, and pH were the abiotic factors altering the studied communities. Surprisingly, the diversity of phytoplankton species did not decrease with increasing temperature, which is inconsistent with the trend observed in permanent water bodies. The study identified specific drivers of phytoplankton community structure and dynamics, underscoring their ecological significance and management implications. These insights will be valuable for future conservation strategies of temporary ponds, crucial in sustaining biodiversity in farmland areas.

**Supplementary Information:**

The online version contains supplementary material available at 10.1038/s41598-025-01060-9.

## Introduction

Kettle holes are small ponds of glacial origin, characteristic of the young moraine landscapes^1^. They differ in shape, size, and hydroperiod (length of the waterphase)^2,3^, as a result they differ much in the fluctuations of the water level^4^. They are widespread in the postglacial region of the northern hemisphere^5–7^, and are often abundant in agricultural landscapes^7,8^.

Isolated ecological wetland islands, such as small ponds, are hotspots of biodiversity in human-transformed landscapes. They provide habitats for a diverse array of species^9,10^, and serve as refugia for terrestrial animals^11^, thus holding high conservation value^12^. They also provide a wide range of ecosystem services, including flood control, improved hydrological cycle, nutrient recycling, toxicant removal, and serving as stepping-stone ecological corridors^13^. Their small volume and placement within farmland areas make them especially susceptible to pollution from the surrounding catchment, resulting in eutrophication and ecosystem degradation^14^. Intensive and destructive land use practices (e.g. drainage of farmland areas, fish stocking, and backfilling of ponds), combined with global warming, pose significant threats to kettle holes and the regional biodiversity they support^12,15^. As a result, in recent years a sharp decline in their number has been observed^8^.

Restoration and creation of ponds are increasingly important measures necessary to mitigate biodiversity loss. To make these actions effective, a thorough understanding of the functioning of these ecosystems is essential. Data on the relationships between the environment and the organisms living in such ponds are largely absent from the literature, particularly studies analyzing long-term trends are lacking. Notably, despite their crucial role in ecosystem functioning, very little is known about the functioning of the phytoplankton communities in such ponds, especially in the temperate climate zone.

Ponds in general seem to offer optimal conditions for algae due to rapid warming of water, good nutrient availability, efficient transport of nutrients to the productive surface zone, and penetration of sunlight into the bottom layers^16^. Thus, species diversity and the number of rare species in small water bodies are usually higher than in other habitat types^17^. Microalgal communities of astatic water bodies are very specific, compared to those of permanent lakes and ponds^18,19^. Due to the great fluctuations of water level and consequently also of physical and chemical variables, they seem to create very unstable conditions for algae. Therefore, phytoplankton of these ecosystems should be dominated by fast-growing, small-sized, and short-lived r-strategists and stress tolerants^4,18^. Following the periodic drying of ponds, a phytoplankton community is reestablished after each dry period, mostly through secondary succession from resting cells preserved in the bottom sediments. The recolonization process after the inundation of the ponds is crucial for the future structure of the communities. Some laboratory studies^19^ showed that diatoms and euglenoids are often the pioneering components of phytoplankton communities in vernal pools, being dominant soon after the inundation. Moreover, euglenoids are able to encyst during unfavorable conditions, thus frequently reaching high abundances and diversity in temporary ponds^20–22^.

Temperature, along with nutrient levels, is a crucial factor in determining the structure of phytoplankton. Due to climate changes and increasing temperature or thermal anomalies, a decrease in phytoplankton diversity is observed in various water bodies throughout the world^23,24^. It is accompanied by higher growth rates and abundance of cyanobacteria, as well as earlier onset and extended duration of cyanobacterial blooms^19,25^. In small water bodies this process is enhanced due to their small depth and volume, and is often accelerating due to the present climate changes.

In ponds located in agricultural areas, nutrient concentrations are relatively high due to both the influx from human altered catchments and significant internal loading from bottom sediments^26,27^. Especially internal supply with phosphorus is increased when compared to lakes because of the small depth of the ponds^28^, creating good conditions for algae growth and promoting long-lasting and frequent water blooms, decreasing species diversity. As a result, nutrient enrichment can sometimes have a greater impact on phytoplankton composition and cyanobacteria growth than temperature^29,30^.

The structure of the phytoplankton communities is affected not only by abiotic variables but also by primary consumers – filter feeders. Among them, endangered large branchiopod crustaceans from the Anostraca and Laevicaudata orders are unique for temporary waters. They hatch en masse when the ponds are filled with meltwater or rainwater and often reach very high densities, which, coupled with relatively large size (up to 2 cm in case of anostracan fairy shrimps described in the present study) make them an important factor shaping phytoplankton communities. As non-selective generalists, they have the potential to significantly lower algal biomass, promoting a clear water state^31^. Under laboratory conditions, fairy shrimps exhibit superior efficacy in controlling cyanobacteria compared to cladocerans^32^. Laboratory experiments on food preferences of one of them – *Eubranchipus grubii* (Dybowski, 1860) showed that it is a nutritional generalist, effectively removing algae from all species present in the water column and potentially shaping their communities^18^. This particular anostracan is relatively common in vernal pools of Central Europe, often occurring in temporary kettle hole ponds^33^. However, despite the laboratory data mentioned above, very little is known about the influence of *E. grubii* – or any other large branchiopod crustaceans – on phytoplankton communities under natural conditions.

Thus, the aim of our study was to analyze changes in phytoplankton structure along the hydroperiod of ponds over a cycle of waterphases during a three years to determine the influence of biotic (large filter feeders) and abiotic (physical and chemical parameters of water) factors. We focused on kettle hole ponds in a farmland of a temperate climate zone – understudied and severely threatened by climate change. We measured phytoplankton species diversity and abundance on three taxonomic levels (dominant taxa, larger taxonomic groups, and total community) and related it to biotic and abiotic factors recorded during our samplings. Based on previous studies^18,19^ our hypothesis was that the large filtrators and abiotic factors would significantly influence the structure and dynamics of the phytoplankton community: phytoplankton diversity would decrease with increasing temperature but the abundance of cyanobacteria would increase; temporal changes in communities will follow shifts in physical and chemical parameters of water; large filter feeders would significantly reduce the abundance of phytoplankton and affect their community structure. Moreover, we hypothesized that diatoms and euglenoids would initiate a secondary succession of phytoplankton right after inundation, and euglenoids would dominate the communities.

## Materials and methods

### Study area

The study covered nine ponds located in postglacial depressions situated in an agricultural landscape of the Wielkopolska province (West Poland), Table 1. The region is located in the temperate climate zone with regular changes in weather and relatively low annual and summer precipitation compared to the other regions of the country^18^. Over the past several decades, the number of small water bodies in this area has decreased due to the intensive use of agricultural land and global warming. The studied pools were typical kettle-shaped pond water bodies, fed mainly by water surface runoff during snow melt in early spring (vernal pools), with the duration of the hydroperiod (5–12 months) depending mainly on the thickness of winter snow cover^34^. Their surface area varies from 189 to 1171 m^2^ and maximum depth from 0.5 to 1.2 m, Table 1.

**Table 1:** Geographical coordinates of the ponds and their maximum depth and surface area.

Pond	Latitude (*N*)	Longitude (E)	Maximum depth [m]	Surface area [m^2^]	Number of samples
BRE	52°29′24″	16°37′37″	1.0	1100	51
BRW	52°29′24″	16°36′05″	1.2	1171	57
DRM	52°28′47″	16°37′09″	0.5	189	39
DRD	52°28′50″	16°37′09″	0.5	401	40
IRS	52°29′31″	16°36′22″	0.7	371	58
STR	52°28′52″	16°36′30″	0.5	877	39
TRI	52°29′04″	16°36′41″	1.0	496	37
TPS	52°29′05″	16°37′09″	0.7	737	16
TPG	52°29′01″	16°37′07″	0.7	593	23

Two ponds (BRW and IRS) did not dry completely during the research period, changing their area to approximately 30% during the summer months (July, August). Other kettle holes dried at least once at different times, from late spring to early autumn. The ponds remained dry from 2 to 9 months.

### Field sampling

Samples for phycological, chemical, and zoological (macroinvertebrates) analyses were collected biweekly from the central area of each pond, beginning at inundation and continuing until the ponds dried. If the ponds didn’t dry, they were sampled until the next spring period. In total, 360 samples were collected for phycological analyzes in from 9 ponds, during 118 weeks (February 2008 to May 2010). The ponds were named using acronyms created basing on the names of closest villages or other topographic features and are also used in our previous papers on ponds from the same region, e.g^34,38^. The number of samples taken from each pond varied depending on the hydroperiod length, see Table 1. For quantitative analyses of phytoplankton, the material was sampled using a calibrated vessel (1 L) and fixed with Lugol solution. For qualitative analyses, samples were collected using a plankton net (mesh size: 25 μm).

Presence and numbering of two large branchiopod crustaceans: anostracan *Eubranchipus grubii* (Dybowski, 1860) and laevicaudatan *Lynceus brachyurs* O.F.Müller, 1776 was assessed in the field each time after the samples were taken. We used a hand net (diameter 30 cm, mesh size 1 mm) towed for a length of 1 m, 30 times in random places covering the area of each pond. The animals were counted each time the net was towed and returned to the pond after each catch. Water temperature, pH, dissolved oxygen, and conductivity were measured directly in the field using a portable multiparameter meter (Hach HQ30d).

### Laboratory analyses

Phytoplankton samples for quantitative analyzes were sedimented in the laboratory and concentrated to a volume of 5–10 ml. The composition and abundance of microalgae were determined with a light microscope (with a magnification of 200x, 400x, and 1000x). The number of algae individuals was counted in at least 400 fields of a Fuchs-Rosenthal chamber (height: 0.2 mm, area: 0.0625 mm^2^). Algal single cells, colonies, coenobia and filaments were treated as individual units. For filaments or trichomes a length of 100 μm was set as one individual.

The diversity index H’ was calculated using the PAST program (http: folk.uio.no/ohammer/past/). It was expressed with the Shannon-Wiener Diversity Index formula^35^. This index unites information on species variety as well as on the relative distribution of species abundance.

The dominating species among phytoplankton were calculated as those which exceeded 10% of the total phytoplankton abundance in each pond and with high frequency (occurring in more than 12% of all samples). Phytoplankton species names were given according to classifications used in Algaebase^36^. Chemical analyzes of water were conducted in the laboratory following standard methods^37^ in order to determine total phosphorus (TP), soluble reactive phosphorus (SRP), nitrate nitrogen (NO_3_-N), nitrite nitrogen (NO_2_-N) and ammonia nitrogen (NH_4_-N) (for details referring to nutrient analyses see^38^).

### Statistical analyses

Analyses were performed on community data that contained abundances of eight taxonomic groups and 33 dominant taxa. Nine environmental variables (water conductivity, pH, NH_4_-N, NO_2_-N, NO_3_-N, SRP, TP, O_2_, temperature), and four biotic variables (presence/absence and numbering of filter feeders: *E. grubii* and *L. brachyurus*) were used as explanatory variables. Variables containing consecutive number of a given sampling event and pond acronym were used as grouping variables for defining random effects or time changes in the analyses (see supplementary material for the data used in all the analyses). To check for changes in phytoplankton over the course of the hydroperiod, a second time variable was introduced, which coded consecutive sampling events within each hydroperiod (thus allowing to nest many hydroperiods within each pond).

To test the hypothesis that euglenoids dominate algal communities a Generalized Linear Mixed-Effects Model (GLMM) was used^43^. The abundance of each taxonomic group in the sample was used as a dependent variable, the group name as a grouping fixed factor and the pond acronym, as well as the sampling date as random effects. The Poisson distribution with logarithmic link was assumed in these analyses. Tukey post hoc test was used to compare the numbering of each pair of taxonomic groups after GLMM was performed. Similar analyses were performed to test if: the values of Shannon index depend on temperature; abundance and dominance of euglenoids and diatoms changes with time; total abundance of algae depends on numbering or presence of *E. grubii* or *L. brahyurus*; abundance and dominance of cyanobacteria changes with water temperature.

The hypothesis that large filter feeders influence algal communities was additionally tested using Analysis of Similarities (ANOSIM) and Similarity Percentages (SIMPER)^44^. A matrix containing the abundance of each phytoplankton group in the samples was used as dependent community dataset and the presence of *E. grubii* or *L. brahyurus* was introduced as the grouping variable. The analyzes were stratified by the variable coding localities sampled. Similar analyzes were repeated to check for influence of large branchiopod presence on abundances of all dominant taxa.

To check what was the response in the structure of algal communities to the nine measured environmental variables describing parameters of water, we used ordination techniques based on direct gradient analyses. Since the community gradient lengths were relatively long (3.768 to 4.334 SD) Canonical Correspondence Analysis (CCA) was chosen^44^. We analyzed the response of the communities at two levels: first, a matrix containing abundances of taxonomic groups (*n* = 8) was used as set of dependent variables. Later, it was replaced by a matrix with abundances of all dominant taxa (*n* = 33) in the second set of analyzes. In both cases, the nine environmental variables were initially introduced as explanatory variables and then subjected to the forward selection test (999 permutations) based on the adjusted R2 and P values^39,44^. The final models presented in the present paper contain only these explanatory variables which significantly improved the model according to the permutation test^44^. To check what were the temporal changes of the phytoplankton communities in response to environmental variables, the same analyzes were later repeated, but this time we used as explanatory variables the interaction terms between the nine environmental factors and the variable describing time after inundation.

All statistical analyzes were performed in R 4.4.1^40^ under RStudio 2024.04.2 Build 764. The following packages were used: ‘multcomp’^41^, ‘MASS’^42^, ‘lme4’^43^ and ‘vegan’^44^. The CCA diagrams were plotted using ‘ggplot’^45^, ‘ggfortify’^46^ and ‘ggrepel’^47^ packages. We considered p = 0.05 as a threshold that determines statistical significance in all analyzes.

## Results

### Phytoplankton species richness and frequency

**Fig. 1:**
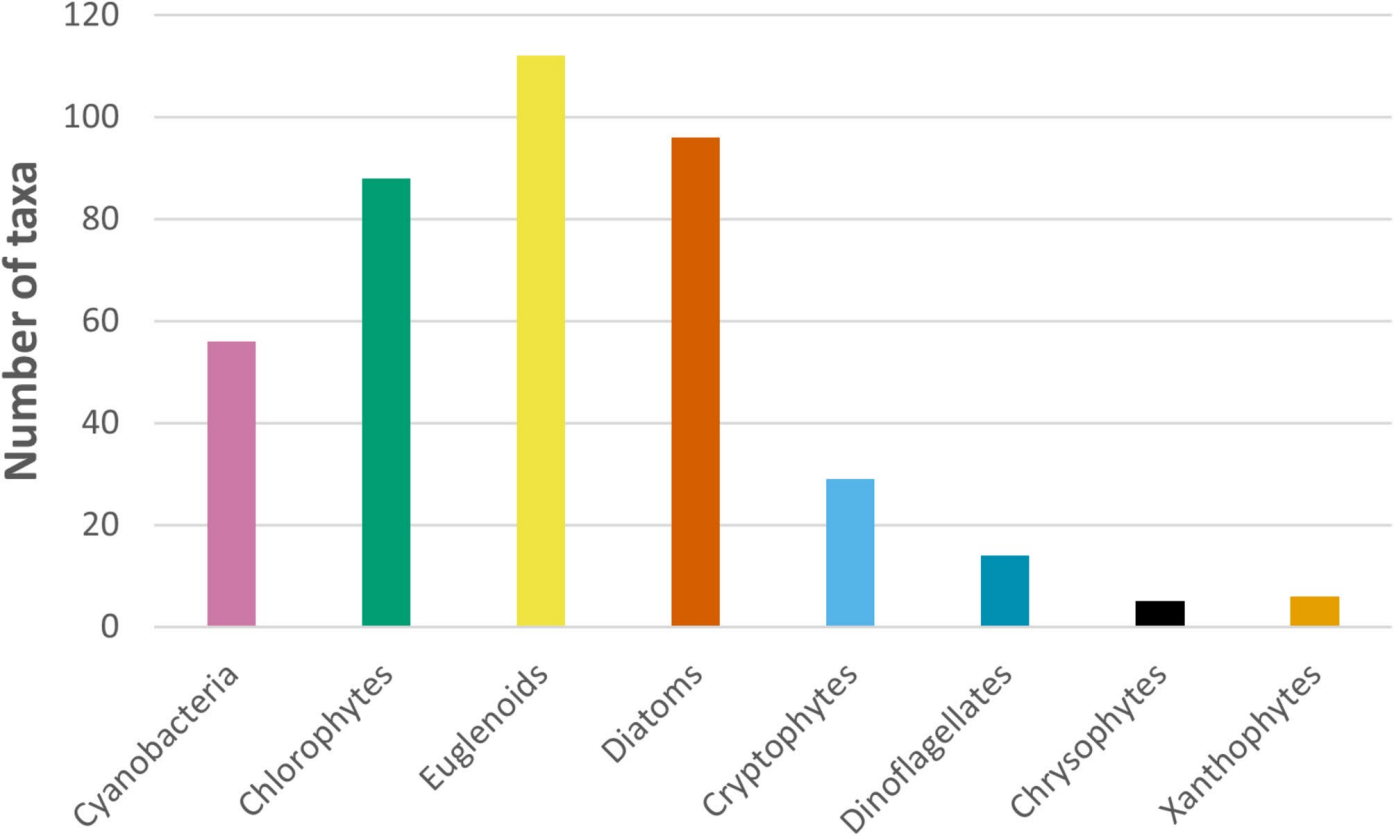
Number of taxa within the particular phytoplankton taxonomic groups in the investigated ponds.

In the nine ponds investigated, 406 phytoplankton taxa have been identified in total, representing 8 systematic groups: euglenoids (112 taxa), diatoms (96), chlorophytes (88), cyanobacteria (56), cryptophytes (29), dinoflagellates (14), xanthophytes (6) and chrysophytes (5), Fig. 1. Euglenoids, diatoms, and chlorophytes had the highest contribution in the total number of taxa (28, 24 and 22%, respectively), compared to the other phytoplankton groups.

The list of phytoplankton species and the frequency of each taxon from all the studied ponds are summarized in Supplementary Material S1. Among the euglenoids, representatives of the genus *Trachelomonas* prevailed and accounted for 42% of the total number of taxa from this systematic algae group. Other euglenoid species belonged to the genera *Astasia*,* Colacium*,* Cryptoglena*,* Discoplastis*,* Euglena*,* Euglenaformis*,* Euglenaria*,* Lepocinclis*,* Menoidium*,* Monomorphina*,* Phacus* and *Strombomonas.*

The taxa most frequently observed in the analyzed ponds were representatives of euglenoids (*Euglena texta*, *Trachelomonas hispida*, *T. intermedia*, *T. volvocina* var. *derephora* and *T. volvocinopsis*), diatoms (*Nitzschia palea*,* Eunotia bilunaris*,* Pinnularia viridis*,* Stauroneis phoenicentron*) and cryptophytes (*Cryptomonas erosa* and *C. marssonii*), which were present at least in 28% of the samples studied (in a minimum of 100 samples), Supplementary Material S1.

### Phytoplankton abundance and diversity

The total phytoplankton abundance in the investigated ponds ranged from 4 to 128 040 indiv. mL^− 1^ during the study period (Supplementary Material S2). The highest abundance was observed during late autumn and winter in the BRW, TPS, and IRS ponds (Supplementary Material S2, Table 2). In this period we recorded high abundance and the large share of chlorophytes (in the BRW and TPS ponds: 111 168 indiv. mL^− 1^ (87%) and 97 896 indiv. mL^− 1^ (99.7%), respectively) and chrysophytes (in the IRS pond: 87 584 indiv. mL^− 1^, 98.9% of the phytoplankton community) (Supplementary Material S3). The mean values of the total abundance ranged from 1 264 indiv. mL^− 1^ to 9 746 indiv. mL^− 1^.

The values of the Shannon-Wiener (H’) species diversity index ranged from 0.01 to 2.86 in the investigated ponds. The mean values were similar in each water body and were usually close to 1 (Table 2; see Supplementary Material S4 for detailed data).

**Table 2:** Total phytoplankton abundance, species diversity Shannon-Wiener’s indices and their minimum (Min), maximum (Max), mean values and standard deviations (SD) in the investigated ponds.

Pond	Total phytoplankton abundance [x10^3^ indiv. mL^− 1^]				Shannon-Wiener diversity index			
	Min	Max	Mean	SD	Min	Max	Mean	SD
BRE	0.01	34.1	3.0	5.9	0.02	2.30	1.13	0.49
BRW	0.036	128.0	9.7	25.1	0.03	2.62	1.34	0.71
DRM	0.006	22.6	1.2	3.8	0.35	2.81	1.40	0.53
DRD	0.016	37.6	4.0	7.5	0.05	2.33	1.18	0.63
IRS	0.011	88.5	5.5	13.2	0.01	2.86	1.00	0.68
STR	0.015	11.3	1.3	2.8	0.16	2.50	1.33	0.63
TRI	0.010	34.0	2.2	6.2	0.02	2.31	1.15	0.57
TPS	0.025	98.2	8.4	25.0	0.13	2.74	1.51	0.72
TPG	0.004	83.9	7.7	18.6	0.02	2.11	0.97	0.60

The most common and abundant phytoplankton groups in all investigated ponds during the study period were chlorophytes, euglenoids, and cryptophytes (Supplementary Material S3). These groups, along with diatoms, were usually the dominant, reaching up to 100% of the total phytoplankton abundance.

Euglenoids were significantly less numerous than chlorophytes (z = -444.42; *p* < 0.0001), which were dominant, significantly outnumbering all other groups (z > 266.82; *p* < 0.0001) in all investigated ponds during the study period. However, euglenoids were the second most dominant group, significantly more numerous than all others (z > 130.34; *p* < 0.0001 in all pair comparisons), except for chlorophytes. Euglenoids (t = -2.164, *p* = 0.0314) and cryptophytes (t = 4.273, *p* < 0.0001) were the only groups, whose relative abundance (%) changed significantly over the course of hydroperiod. The contribution of euglenoids decreased, whereas the share of cryptophytes increased. The share of diatoms did not change significantly over the course of hydroperiod (t = -0.898, *p* = 0. 0.37).

Among the seven ponds that dried during the research period, most of them (STR, DRD, DRM, TRI, TPG) were mainly dominated by diatoms (55% of the total phytoplankton abundance in STR pond, 53% in DRM, 96% in TRI, and 77% in TPG) and euglenoids (96% in DRD, 38% in DRM, 77% in TPG), which initiated the secondary succession of phytoplankton immediately after inundation (Supplementary Material S3). In contrast, in the BRE and TPS ponds, chlorophytes (mainly from the genus *Chlamydomonas*) dominated after the hydroperiod started, with their share in the total phytoplankton abundance being 99% and 66%, respectively.

### Relationships between phytoplankton abundance and biotic (large filter feeders) and abiotic factors

According to statistical analyzes, the numbering of large branchiopod filter feeders (*E. grubii* and *L. brachyurus*) did not affect the total abundance of phytoplankton (t = -0.044; *p* = 0.9647 and t = 1.215; *p* = 0.2253, respectively) in the investigated ponds during the study period. However, according to ANOSIM, the presence of each filter feeder species was related to changes in the structure of the microalgal community (*R* = 0.1023; *p* = 0.004 and *R* = 0.08748; *p* = 0.021, respectively). SIMPER analysis showed that the primary taxonomic groups contributing to the difference were chlorophytes, euglenoids, and cryptophytes. However, only in the case of chlorophytes these differences in abundance between samples with and without large branchiopods were significant (*E. grubii*: cusum = 0.352; *p* = 0.002; *L. brachyurus*: cusum = 0.343; *p* = 0.005), with higher abundances of chlorophytes coinciding with the presence of filter feeders.

**Fig. 2:**
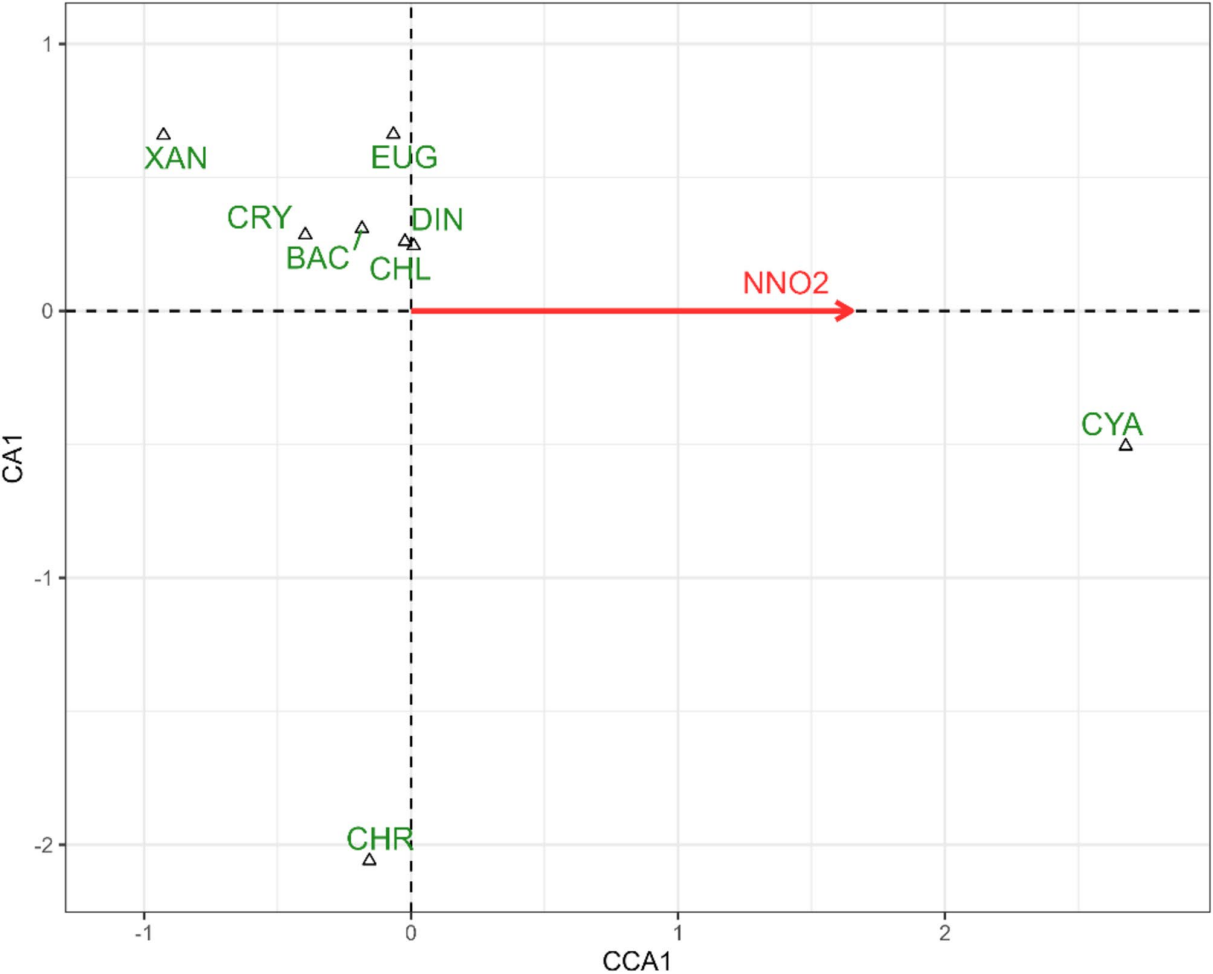
Diagram of Canonical Correspondence Analysis (CCA) illustrating the distribution of phytoplankton taxonomic groups in relation to environmental variables recorded during the samplings. Only one explanatory variable was significant according to permutation test, thus this is the only one included in the model and displayed on the diagram. Abbreviations: CYA – Cyanobacteria; CHL – chlorophytes; BAC – diatoms; XAN – xanthophytes; CHR – chrysophytes; EUG – euglenoids; DIN – dinoflagellates; CRY – cryptophytes; NNO2 (NO2-N) – nitrite nitrogen.

In the CCA model checking for the influence of abiotic factors on the structure of phytoplankton communities at the level of eight taxonomic groups, only the concentration of NO_2_ was significant (F = 27.26; *p* = 0.011) and was positively correlated with the abundance of cyanobacteria (Fig. 2). The remaining phytoplankton groups were negatively related to nitrites. The model was significant at F = 27.26, *p* = 0.006, variation explained: 26.1%, eigenvalue of the first (and the only constrained) axis: 0.24711.

**Fig. 3:**
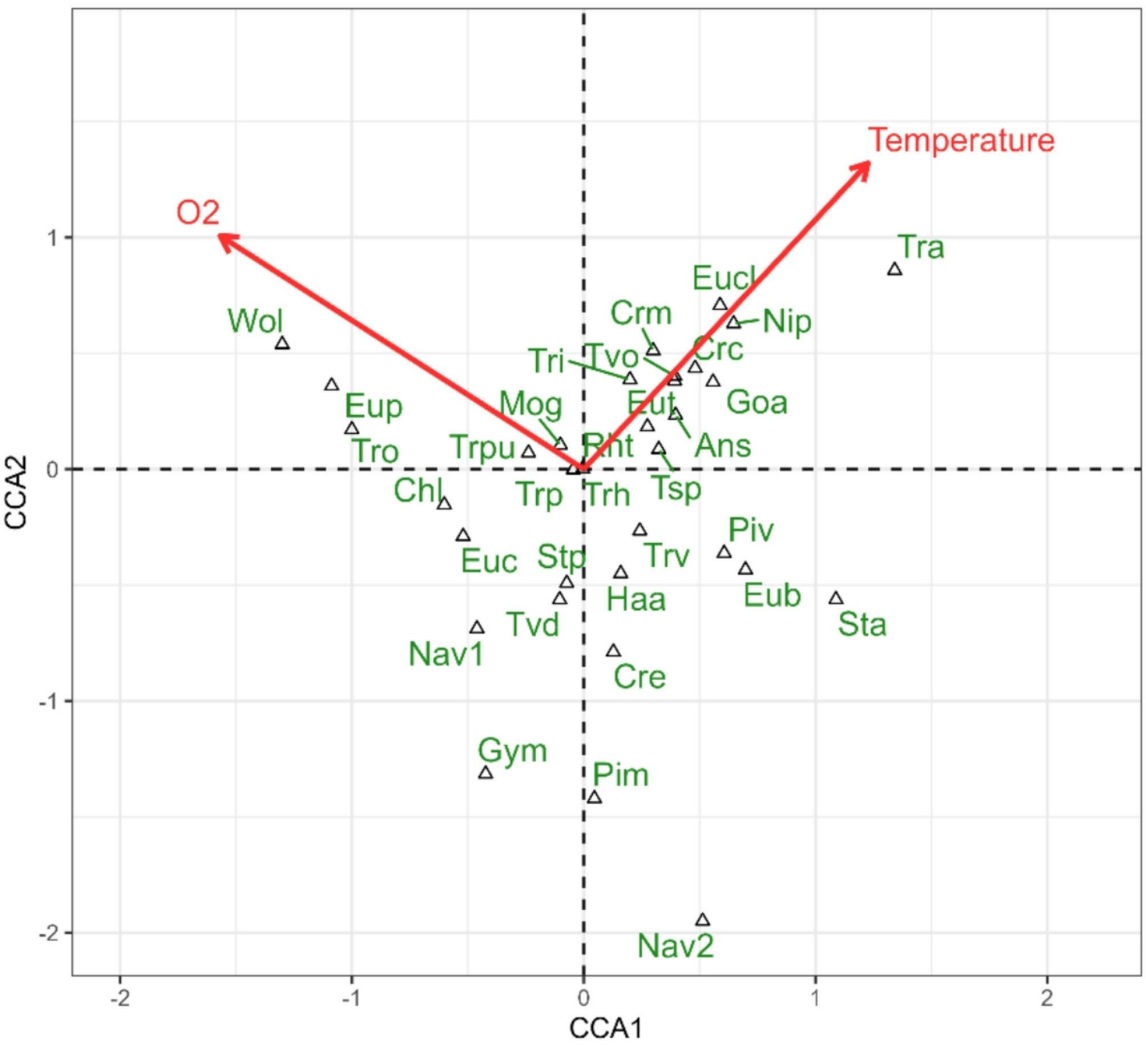
Diagram of Canonical Correspondence Analysis (CCA) illustrating the distribution of phytoplankton taxa in relation to environmental variables recorded during the samplings. Only the explanatory variables significant according to permutation test are included in the model and displayed on the diagram. Abbreviations: chlorophytes: Ans – Ankistrodesmus stipitatus; Chl – Chlamydomonas sp.; Mog – Monoraphidium griffithii; euglenoids: Euc – Euglenaria caudata; Eucl – Euglenaria clavata; Eup – Euglenaformis proxima; Eut – Euglena texta; Tra – Trachelomonas armata; Trh – Trachelomonas hispida; Tri – Trachelomonas intermedia; Tro – Trachelomonas oblonga; Trpu – Trachelomonas oblonga var. pulcherrima; Trp – Trachelomonas pusilla; Trv – Trachelomonas volvocina; Tvd – Trachelomonas volvocina var. derephora; Tsp – Trachelomonas sp.; Tvo – Trachelomonas volvocinopsis; diatoms: Eub – Eunotia bilunaris; Goa – Gomphonema augur; Haa – Hantzschia amphioxys; Nav1 – Navicula sp. 1; Nav2 – Navicula sp. 2; Nip – Nitzschia palea; Pim – Pinnularia mesolepta; Piv – Pinnularia viridis; Sta – Stauroneis anceps; Stp – Stauroneis phoenicenteron; cryptophytes: Cre – Cryptomonas erosa; Crm – Cryptomonas marssonii; Crc – Cryptomonas curvata; Rht – Rhodomonas tenuis; dinoflagellates: Gym – Gymnodinium sp.; Wol – Woloszynskia sp.; Temp – water temperature; O2 – dissolved oxygen contents.

At the level of the dominant taxa composition, the CCA model showed that the water temperature and dissolved O_2_ content significantly shaped the algal community (F = 7.7041; *p* = 0.031 and F = 8.6301; *p* = 0.031, respectively), Fig. 3. Positively related to temperature were abundances of *Euglenaria caudata*, *Euglenaria clavata*, *Euglena texta*, *Trachelomonas armata*, *Trachelomonas intermedia*, *Trachelomonas volvocinopsis*, *Trachelomonas* sp., *Gomphonema augur*, *Nitzschia palea*, *Cryptomonas curvata*, *Cryptomonas marssonii*, *Ankistrodesmus stipitatus* and *Rhodomonas tenuis*, while negatively related were mainly *Chlamydomonas* sp. *Woloszynskia* sp., *Euglenaformis proxima* and *Trachelomonas oblonga* were strongly positively associated with O_2_, whereas *Stauroneis anceps*, *Eunotia bilunaris*, *Pinnularia viridis* and *Trachelomonas volvocina* were negatively related to this variable. The model was significant at F = 5.3855, *p* = 0.033, variation explained: 19.8%, eigenvalue of the first axis: 0.23610, eigenvalue of the second axis: 0.17132.

### Succession in phytoplankton communities related to environmental factors

The rise in the water temperature values in the studied ponds coincided with the increase in total phytoplankton abundance over time (t = − 3.037; *p* = 0.00343). However, the abundance of cyanobacteria did not increase with temperature (t = − 1.085; *p* = 0.2786). Values of Shannon-Wiener species diversity indices did not decrease significantly with increasing temperature (t = − 0.05; *p* = 0.96).

**Fig. 4:**
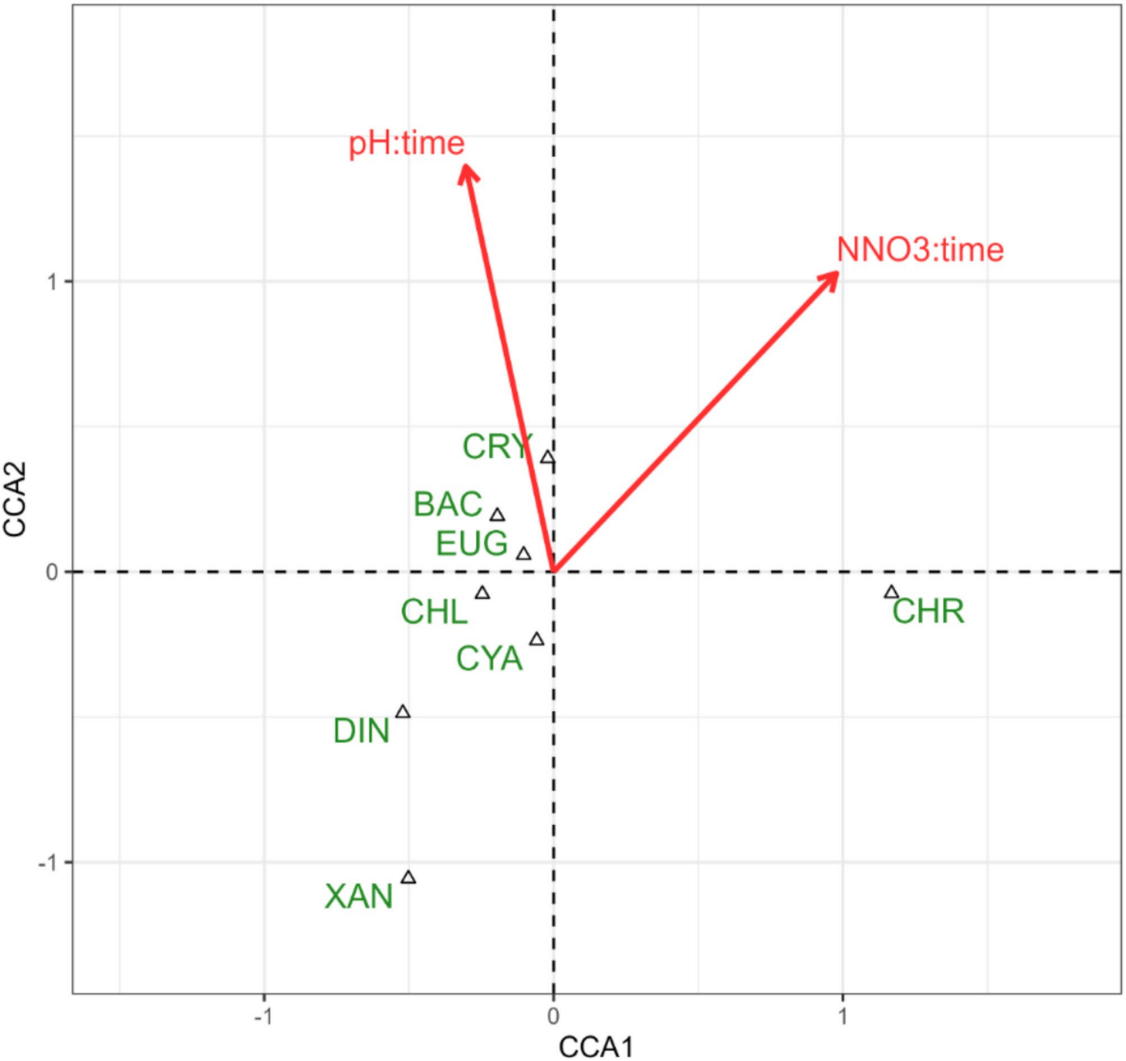
Diagram of Canonical Correspondence Analysis (CCA) illustrating the relation between the changes in abundance of phytoplankton taxonomic groups and changes in environmental variables recorded during the samplings. Only one explanatory variable was significant according to permutation test, thus this is the only one included in the model and displayed on the diagram. Abbreviations: pH; NNO3 (NO3-N) – nitrate nitrogen; other see Fig. 2.

Temporal changes in abundance of the eight taxonomic groups were significantly related to the changes in pH value and NO_3_-N (F = 12.765; *p* = 0.048 and F = 14.303; *p* = 0.039, respectively), Fig. 4. Cryptophyte and diatom abundance was increasing as the values of these parameters increased, while changes in the abundance of dinoflagellates and xanthophytes were negatively related to these two environmental variables. The entire model was significant at F = 13.777; *p* = 0.019, variation explained: 26.2%, the eigenvalue of the first axis was 0.21922, and that of the second axis was 0.03102.

**Fig. 5:**
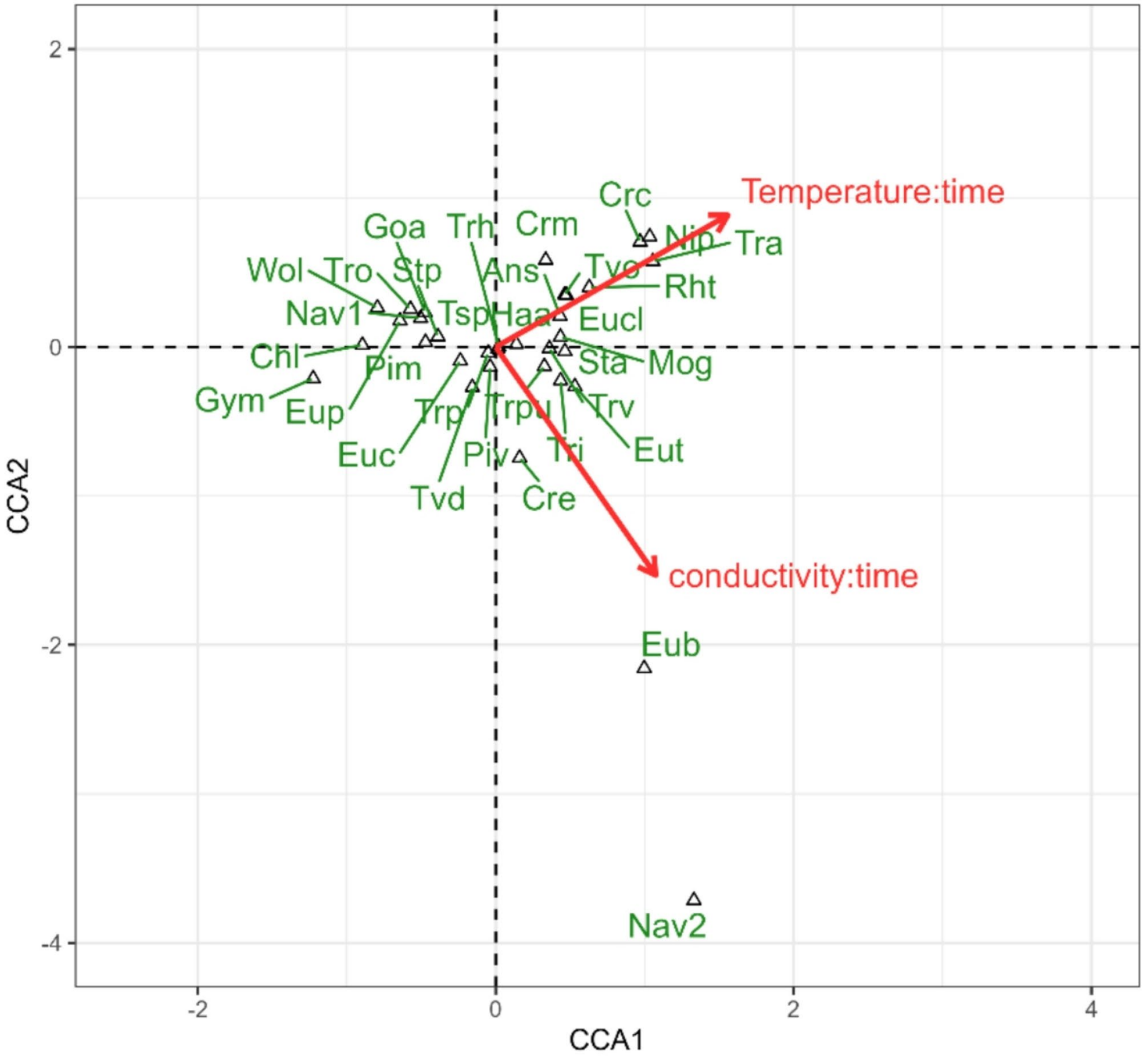
Diagram of Canonical Correspondence Analysis (CCA) illustrating the relation between the changes in abundance of phytoplankton taxa and the changes in environmental variables recorded during the samplings. Only the explanatory variables significant according to permutation test are included in the model and displayed on the diagram.

The CCA model conducted on the level of dominant taxa showed that changes in the structure of the phytoplankton communities were significantly influenced by both temperature (F = 9.7355; *p* = 0.021) and conductivity (F = 8.6779; *p* = 0.039), Fig. 5. According to the results, changes in the abundance only of a few taxa were clearly positively associated with increasing conductivity (diatoms: *Eunotia bilunaris* and *Navicula* sp. 2 and cryptophyte *Cryptomonas erosa*), while changes in abundance of *Woloszynskia* sp., *Euglenaformis proxima*,* Gomphonema augur*, *Stauroneis phoenicenteron*, *Pinnularia mesolepta* and *Trachelomonas oblonga* were negatively affected by this parameter. Increase in abundance of a large group of species was positively related with rising water temperature: *Ankistrodesmus stipitatus*, *Monoraphidium griffithii*, *Nitzschia palea*, *Stauroneis anceps*, *Euglenaria clavata*,* Trachelomonas volvocinopsis*,* Trachelomonas armata*, *Cryptomonas curvata*,* Cryptomonas marssonii* and *Rhodomonas tenuis*. Significance of the whole model: F = 9.3147, *p* = 0.015, variation explained: 20.3%; eigenvalues: axis 1 = 0.25913; axis 2 = 0.19814.

ANOSIM analyses showed, that phytoplankton communities significantly differed on the level of broader taxonomic groups depending on Anostraca presence (*R* = 0.1098; *p* = 0.001). In the presence of filter feeders we observed significantly higher abundances of unicellular chlorophyte *Chlamydomonas* sp. (cumsum = 0.501; *p* = 0.006), diatoms (*Navicula* sp.: cumsum = 0.713; *p* = 0.001 and *Hantzschia amphioxys*: cumsum = 0.967; *p* = 0.041), dinoflagellate – *Gymnodinium* sp. (cumsum = 0.739; *p* = 0.009) and euglenoid – *Trachelomonas oblonga* (cumsum = 0.766; *p* = 0.012).

## Discussion

Phytoplankton species richness in the investigated kettle hole ponds was generally high. The communities were enriched by many tychoplanktonic (non – planktonic) taxa, originally benthic (living on, in, or near the bottom) or periphytic (adopted to firmly attaching to substratum), which accidentally ended up in plankton assemblages. Tychoplanktonic forms are known to be numerous in small and shallow water bodies^48^, where they often become part of the phytoplankton communities as a result of intensive water mixing. Among the originally benthic algae, many species belonging to diatoms (e.g. representatives from the genera *Caloneis*,* Cymbella*,* Navicula*,* Pinnularia*,* Stauroneis*^49^) and chlorophytes (desmids from genera *Closterium* and *Cosmarium*^50^), were found in the phytoplankton communities of the studied ponds. Among originally periphytic taxa, diatoms e.g. from the genera *Achnanthidium*,* Epithemia*,* Eunotia*,* Fragilaria* and *Gomphonema* and cyanobacteria (mainly Oscillatoriales) were noted.

Interestingly, some of the identified species are very rare in Poland or in the world, e.g. chlorophyte *Desmatractum indutum*^51^ or euglenoid *T. sydneyensis*^52^. Three of the diatom taxa are listed on the Polish Red List of Algae^53^ and classified as endangered (in danger of extinction): *Pinnularia nobilis* and *P. schoenfelderii* or vulnerable (believed likely to move into the endangered category in the near future) – *Stauroneis phoenicentron*. This demonstrates that ephemeral habitats generate distinct, unstable environmental conditions for the organisms that inhabit them. These conditions are characterized by significant fluctuations in the physical and chemical parameters of water, which may also promote high species richness^4^ and occurrence of some rare species.

In line with our initial hypothesis, most of the noted phytoplankton taxa – including the most frequent species – belong to euglenoids, which are characteristic for small, shallow and rapidly warming freshwater habitats rich in dissolved organic matter^20,54^, including farmland kettle holes. Due to their ability to form resting stages (cysts) to survive unfavorable conditions like drought period^52^, they are adapted to live also in temporary ponds. On the other hand, a high species richness in the investigated samples was probably also a consequence of favorable environmental conditions prevailing in small water bodies, like rapid heating of water, large quantity and availability of nutrients and favorable light supply. During our field study levels of nutrients were changing from very low to high, which can explain some of the observed changes in phytoplankton communities. Among euglenoids, the most species belonged to the genus *Trachelomonas*, characteristic of eutrophic water environments^55^, including field ponds.

Frequent changes in phytoplankton abundance observed in the investigated kettle holes seem to be justifiable for small and astatic water bodies, as opposed to the more stable conditions found in permanent ponds^56^. This is in agreement with the implications of other studies^57,58^, which also show that the structure of phytoplankton assemblages of small water bodies, especially temporary ones, is characterized by great quantitative and qualitative fluctuations over time. The high variation in environmental factors, both physical and chemical, when compared to larger water bodies can be attributed to the small size and drying cycles of ponds^59^. In the quantitative structure of phytoplankton communities, this causes and favors a high contribution of stress-tolerant, opportunist species (with a low level of specialization, able to live in various habitats and easily adapting to environmental changes). Such species are often small-sized, single-celled, fast-growing r-strategists with short life cycles typical of temporary ponds^4,24^, e.g. cryptophytes and some chlorophytes. Moreover, the frequent domination of euglenoids noted in our study is also known to be characteristic of ephemeral habitats because of their ability to survive in unfavorable environments^52^. Euglenoids also show preferences towards high nutrient concentrations connected with organic pollution in field ponds^60^. Generally, flagellated algae (euglenoids, cryptophytes and some chlorophytes) whose share in the total abundances of microalgae was large in the investigated ponds, are known to be common in small-sized water bodies^61^. Additionally, the high contribution of tychoplanktonic diatoms (mainly originally benthic *Hantzschia amphioxys*,* Navicula* sp., *Nitzschia palea*,* Pinnularia mesolepta*,* P. viridis*,* Stauroneis anceps* and *S. phoenicentron*) in the phytoplankton total abundances in our study is consistent with the results of other studies concerning small water bodies, including field ponds^19^. Following the functional group (FG) classification (after^20^ and reviewed by^62^), the phytoplankton dominating taxa in the investigated kettle holes were characteristic for small, shallow and turbid water bodies rich in nutrients prevailing in the agricultural landscapes (belonging to the groups: MP - genera *Eunotia*,* Gomphonema*,* Navicula*; X1 - *Ankistrodesmus stipitatus*,* Monoraphidium griffithii*; X2 - *Chlamydomonas* sp., *Rhodomonas tenuis*; W1 - genera *Euglena/Euglenaformis/Euglenaria*; W2: representatives of the genus *Trachelomonas*; Y - genera *Cryptomonas*,* Gymnodinium* and *Woloszynskia*). Moreover, one of the diatoms recorded in our study (*Nitzschia palea*) is typical for inorganically turbid shallow lakes and belongs to the functional group D.

Low values of the Shannon-Wiener species diversity index (0.01–2.86) in particular ponds indicate low species diversity and a clear dominance of single species with a simultaneous small share of other taxa, which is usually connected with high fertility in water ecosystems^63^. This could be caused by the agricultural type of the catchment area of the studied kettle holes and external nutrient loading due to the use of fertilizers and pesticides from nearby farmlands. For comparison, the phytoplankton diversity of lakes typically ranges from 2.4 to 2.6^64^, while in the case of other eutrophic small water bodies in agricultural areas in Wielkopolska Province, it is on average about 2^19^. The low diversity index in the investigated ponds can be explained by their astatic character.

Surprisingly, we found that phytoplankton species diversity did not decrease with increasing temperature. This finding is inconsistent with our assumptions and with the findings of many earlier studies^24,65^, which showed that phytoplankton species diversity decreased with higher temperatures and also with climate warming. Meanwhile, according to^66^, temporary and astatic waters are highly susceptible to rapid heating and cooling, even daily or hourly, due to their small area and depth. Such specific thermal conditions do not seem to be significant for phytoplankton diversity, considering that phytoplankton communities of kettle hole ponds mainly consist of species adapted to unstable conditions. This should be important when considering the influence of global warming on the ecosystems of astatic water bodies.

In accordance with our hypothesis, diatoms and euglenoids predominantly initiated the secondary succession of phytoplankton right after inundation in most of the kettle holes with a dry period. Similar observations have been made in laboratory conditions, during experiments in microcosms using bottom sediments from the vernal pool^19^. One of the reasons for diatom and euglenoid initial dominance was probably the fast reproduction rate in the representatives of both taxonomic algae groups^52^, so they are often the winning competitors in aquatic environments^67^. Diatom preferences for cooler waters^68,69^ together with low light intensity and short photoperiod tolerance^70^, additionally explain their initial dominance during the winter months (January and February with short days and low temperatures) in the investigated ponds. Euglenoids, in turn, are known to thrive across a broad spectrum of temperatures^19^. Their large contribution to the phytoplankton communities right after inundation of ponds during periods of shorter daylight (specifically at the end of November and in January), may be attributed to their mixotrophic nutritional strategy^71^. This strategy allows them to sustain themselves independently of light availability^19^. Furthermore, fast-growing r-strategist chlorophytes from the *Chlamydomonas* genus that are able to rapidly incorporate existing resources, also dominated at the beginning of the hydroperiod, but only in the case of two ponds. This is in line with other studies^57,72^, which also prove that species initially abundant as early colonizers are usually small and fast-growing organisms, e.g., unicellular *Chlamydomonas* sp. Moreover, these taxa preferred lower water temperatures prevailing at the beginning of the hydroperiod in the investigated ponds, which was consistent with our CCA analysis. Thus, the results suggest that when the hydroperiod in kettle hole ponds begins during colder times of the year with a short photoperiod, mainly diatoms, euglenoids, or *Chlamydomonas* sp. will initiate the phytoplankton succession and will be successful competitors at the onset of the hydroperiod. This fact is important from the perspective of the functioning of food webs in temporary waters, particularly for the development of large filter feeders (e.g., Anostraca) that feed on microalgae. Most of them – especially anostracan crustaceans – are characterized by periodic mass occurrence, usually at the beginning of the inundation period^4^.

As we expected, large filter feeders (Anostraca and Laevicaudata) significantly affected the phytoplankton community structure, although they did not influence the total phytoplankton abundance. Our results are partially inconsistent with previous studies, which showed a significant reduction of the total phytoplankton abundance caused by large branchiopods (e.g., *Artemia franciscana*, *Branchinecta* sp., *E. grubii*). However, the above-mentioned studies are based on laboratory experiments (e.g^19^). , where the phytoplankton species richness was small compared to those in aquatic ecosystems, or on field investigations (from arid environments of deserts and polar or alpine zones) with the presence of water blooms and the dominance of one to several algae species^73^. On the other hand, there are also reports showing no significant effect of Anostraca (e.g^74^. on *Artemia salina*;^75^ on *Branchinecta gigas* and *B. mackini*) on algal communities in ponds. None of these field studies, however, covered temporary waters in temperate climate zones and there is generally a lack of knowledge on this subject.

According to our findings, the presence of both Anostraca and Laevicaudata was related to a higher abundance of chlorophytes, which are usually highly susceptible to grazing pressure; thus, large filter feeders stimulate their growth and increase their abundances by grazing (especially in fast-growing r-strategist chlorophytes like *Chlamydomonas* sp.;^16^). Chlorophytes (especially the flagellates dominant in our study) are also favored food sources for filter feeders^76^. It is well documented that algae under strong grazing pressure are able to increase their abundance by their rapid growth rates that compensate for increased grazing losses^77^. Filter feeders may also increase the abundance of chlorophytes indirectly by the resupply of nutrients (through excretion) which are essential for phytoplankton development^78^. On the other hand, the high abundance of chlorophytes could be the factor that caused higher survivability of the selective grazers at their larval stages, thus leading to higher crustacean abundances. This could be particularly true in the case of some other dominating small and unicellular phytoplankton taxa, such as representatives of diatoms, euglenoids, and dinoflagellates, which were also associated with large filter feeders.

Among abiotic factors significantly influencing the quantitative structure of phytoplankton communities, pH, NO_3_-N and NO_2_-N, conductivity, and water temperature were noted. Changes in the abundance of cryptophytes and diatoms were associated with nitrate nitrogen, Diatoms are known to be well adapted to use specifically NO_3_-N^79^, which is consistent with our results. Cryptophytes, however, are generally known to prefer NH_4_-N^79^. On the other hand, the negative correlations observed between the abundance of certain microalgae (e.g. cyanobacteria, dinoflagellates, and xanthophytes) and NO_3_-N over time in our study may result from their ability to remove nitrogen pollutants from ponds through efficient nutrient uptake, which was observed also by some authors^80^.

The increase in temperature positively affected the dynamics of the total phytoplankton abundance and the abundance of most species dominating in the studied ponds (especially representatives of euglenoids, cryptophytes, and some chlorophytes), both over time and excluding the time factor. On the other hand, most of the diatom-dominating species in our study (e.g. *Stauroneis phoenicentron*,* Pinnularia mesolepta*,* P. viridis*) preferred cooler waters. These findings are mostly consistent with the results of other studies: chlorophyte species are usually associated with increasing water temperature^69,81^, and diatoms in general tend to dominate in lower temperatures^82,83^. Mixotrophic flagellate species are able to grow well during the colder months of the year in lakes in temperate zones^83^, which is in line with our results regarding dinoflagellates (*Gymnodinium* sp. and *Woloszynskia* sp.), but not in case of euglenoids (e.g. *Euglenaria clavata*,* Trachelomonas armata*,* T. volvocinopsis* and *Trachelomonas* sp.) and cryptophytes (*Cryptomonas curvata*,* C. marssonii*,* Rhodomonas tenuis*). In the studied kettle hole ponds these species were clearly associated with higher temperatures or neutral in relation to temperature.

Contrary to our predictions, cyanobacteria of the investigated kettle holes did not favor high temperatures. This finding contrasts with their documented preference for warm waters^69^ and their tendency to cause blooms primarily during summer in surface waters of temperate climate zones. Our findings were consistent, however, with the previous results from a laboratory experiment using sediments from a vernal pool^19^, in which cyanobacteria preferred low (4 °C) or moderate (16 °C) temperatures. Thus, temporary kettle hole ponds appear to be inhabited by specific cyanobacterial species that prefer lower water temperatures, likely due to the large daily temperature fluctuations and frequent low night temperatures in these astatic habitats. Additionally, other conditions typical of such water habitats can be unfavorable for cyanobacteria and inhibit their population growth. Known factors include significant fluctuations in water levels, frequent water mixing, and high variation in other abiotic parameters^4^. Most cyanobacteria prefer stable environmental conditions with minimal water mixing^84^. Consequently, cyanobacteria appear to be outcompeted by other microalgae typical of small water bodies, like euglenoids, small chlorophytes, cryptophytes, and diatoms^85^. However, as demonstrated by^86^, the effects of warming and temperature can vary even between cyanobacteria genera. According to our study, specific cyanobacteria species that tolerate unstable environmental conditions of kettle hole ponds are primarily colonial coccoid genera, such as *Rhabdogloea* and *Chroococcus*. These taxa occasionally played a significant role in the phytoplankton communities of the studied kettle hole ponds, with their highest abundance observed during the cooler months of spring and autumn (March–May and October–November).

Our study showed some specific and significant relationships between phytoplankton and environmental factors existing in poorly investigated habitats of temporary kettle hole ponds. Both biotic (large filter feeders) and abiotic factors (especially nitrogen forms, pH, water temperature, and conductivity) turned out to be particularly important in shaping algae communities. Detailed analyses suggest food preferences of large filter feeders both in relation to individual species and to some taxonomic groups of algae (especially chlorophytes). Moreover, the significant influence of specific physical and chemical parameters of water on dominant phytoplankton species, groups, and biodiversity has also been demonstrated. Thus, our results indicated the important drivers of the phytoplankton community structure and dynamics in temporary kettle holes, differing substantially from those in permanent lakes and ponds.

In the context of climate change projections, our findings indicate that the contribution of cyanobacteria to phytoplankton communities in kettle hole ponds should not increase with rising temperatures. Furthermore, there should be no decline in species diversity, in contrast to permanent standing water bodies. Additionally, we do not anticipate a deficit in the food source for large filter feeders, which are unicellular green algae. Rising temperatures will, however, significantly change the structure of planktonic communities, primarily reducing the occurrence of cold-water species. Additionally, changes in hydrology due to varying precipitation patterns will undoubtedly impact the functioning of algal communities, although this aspect was not within the scope of our study.

The results of our study will contribute to a better understanding of phytoplankton community dynamics in temporary aquatic ecosystems and the role of microalgae as bioindicators. They also shed light on the functioning of kettle hole pond ecosystems, their biodiversity, and ecological value, aiding in their management and protection. Understanding the influence of macroinvertebrates and environmental factors on phytoplankton communities in ponds is essential for advancing ecological research, conservation, and monitoring in the era of global climate change, due to their fast responses to environmental changes. Most of all, however, our study underlines the fundamental importance of temporary waters as local biodiversity hotspots, hosting high species richness and rare phytoplankton taxa.

## Electronic supplementary material

Below is the link to the electronic supplementary material.

Supplementary Material 1

Supplementary Material 2

Supplementary Material 3

Supplementary Material 4

## Data Availability

All data collected and analyzed during this study are included in this published article (and its supplementary files).
