# Supplementary material for: Shifts in phytoplankton communities in response to water parameters and large branchiopod filter feeders in kettle hole ponds of farmland landscape
Source: Sci Rep. 2025 May 21;15:17623. doi: 10.1038/s41598-025-01060-9 (PMC12095657; doi:10.1038/s41598-025-01060-9)
Supplement: Supplementary file 1 — Supplementary Material 1 [file 41598_2025_1060_MOESM1_ESM.docx]

**Supplementary Material S1.** List and frequency of phytoplankton taxa found during the study period in the investigated ponds.

|  |  |
| --- | --- |
| **Cyanobacteria** | **Frequency (%)** |
| *Anabaena oscillarioides* Bory ex Bornet & Flahault | 4.5 |
| *Anabaena sp.* | 1.9 |
| *Aphanizomenon flos-aquae* Ralfs ex Bornet & Flahault | 0.6 |
| *Aphanizomenon sp.* | 0.3 |
| *Aphanocapsa grevillei* (Berkeley) Rabenhorst | 1.9 |
| *Aphanocapsa* sp. | 0.6 |
| *Aphanothece microscopica* Nägeli | 0.3 |
| *Aphanothece stagnina* (Sprengel) A.Braun | 0.6 |
| *Aphanothece* sp. | 0.8 |
| *Chamaesiphon confervicola* A. Braun | 5.55 |
| *Chamaesiphon cylindrosporus* Skuja | 0.6 |
| *Chamaesiphon* sp. | 5.0 |
| *Chroococcus dispersus* (Keissler) Lemmermann | 0.6 |
| *Chroococcus minutus (*Kützing) Nägeli | 0.3 |
| *Chroococcus turgidus* (Kützing) Nägeli | 1.4 |
| *Chroococcus* sp. | 1.4 |
| *Cuspidothrix issatschenkoi* (Usachev) P. Rajaniemi Komárek R.Willame P. Hrouzek K.Kastovská L.Hoffmann & K.Sivonen | 0.3 |
| *Cylindrospermum* sp. | 1.0 |
| *Dolichospermum affine (Lemmermann)* Wacklin L. Hoffmann & Komárek | 4.0 |
| *Dolichospermum circinale* (Rabenhorst ex Bornet & Flahault) Wacklin Hoffmann & Komárek | 0.3 |
| *Dolichospermum spiroides*(Klebhan) Wacklin L. Hoffmann & Komárek | 0.3 |
| *Gomphosphaeria* sp. | 0.3 |
| *Hyella* sp. | 1.0 |
| *Jaaginema pseudogeminatum* (G. Schmid) Anagnostidis & Komárek | 0.8 |
| *Johannesbaptistia lacustris* Hindák | 0.3 |
| *Leptolyngbya thermalis* Anagnostidis | 0.8 |
| *Limnococcus limneticus* (Lemmermann) Komárková Jezberová O.Komárek & Zapomelová | 1.7 |
| *Limnoraphis hieronymusii* (Lemmermann) J. Komárek E. Zapomelová J. Smarda J. Kopecký E. Rejmánková J. Woodhouse B. A. Neilan & J. Komárková | 0.3 |
| *Limnothrix lauterbornii* (Schmidle) Anagnostidis | 0.6 |
| *Limnothrix redekei* (Goor) Meffert | 3.9 |
| *Lyngbya* sp. | 0.3 |
| *Merismopedia tranquilla* (Ehrenberg) Trevisan | 0.8 |
| *Microcystis aeruginosa* (Kützing) Kützing | 0.8 |
| *Microcystis marginata* (Meneghini) Kützing | 0.3 |
| *Microcystis viridis* (A. Braun) Lemmermann | 0.3 |
| *Microcystis wesenbergii* (Komárek) Komárek ex Komárek | 4.5 |
| *Microcystis* sp. | 0.6 |
| *Nodularia* sp. | 0.8 |
| *Oscillatoria tenuis* C. Agardh ex Gomont | 0.3 |
| *Oscillatoria* sp. 1 | 9.4 |
| *Oscillatoria* sp. 2 | 7.2 |
| *Phormidium granulatum* (N. L. Gardner) Anagnostidis | 0.3 |
| *Phormidium terebriforme (C. Agardh ex Gomont) Anagnostidis & Komárek* | 1.9 |
| *Phormidium* sp. | 4.5 |
| *Planktolyngbya contorta* (Lemmermann) Anagnostidis & Komárek | 1.7 |
| *Planktothrix agardhii* (Gomont) Anagnostidis & Komárek | 2.0 |
| *Pseudanabaena limnetica* (Lemmermann) Komárek | 2.8 |
| *Pseudanabaena* sp. | 0.3 |
| *Raphidiopsis raciborskii* (Woloszynska) Aguilera & al. | 0.3 |
| *Rhabdogloea smithii* (Chodat & F. Chodat) Komárek | 2.5 |
| *Rhabdogloea linearis* (Geitler) Komárek | 0.8 |
| *Romeria chlorina* Böcher | 0.8 |
| *Snowella* sp. | 0.6 |
| *Spirulina* sp. | 0.8 |
| *Woronichinia* sp. 1 | 0.6 |
| *Woronichinia* sp. 2 | 1.0 |
| **Chlorophytes** |  |
| *Actinastrum hantzschii* Lagerheim | 1.0 |
| *Ankistrodesmus falcatus* (Corda) Ralfs | 2.0 |
| *Ankistrodesmus stipitatus* Komárková-Legnerová | 17.8 |
| *Ankyra judayi* (G. M. Smith) Fott | 0.3 |
| *Botryococcus braunii* Kützing | 0.6 |
| *Bulbochaete* sp. | 0.3 |
| *Characium angustum* A. Braun | 5.55 |
| *Characium* sp. | 1.7 |
| *Chlorogonium elongatum* var. *aculeatum* (Pascher) L. Péterfi | 9.2 |
| *Chlamydomonas globosa* J. W. Snow | 10.8 |
| *Chlamydomonas microscopica* G. S. West | 5.0 |
| *Chlamydomonas* sp. 1 | 9.2 |
| *Chlamydomonas* sp. 2 | 15.3 |
| *Chlamydomonas spinifera* Ettl | 0.6 |
| *Chlorogonium* sp. 1 | 6.7 |
| *Chlorogonium* sp. 2 | 5.3 |
| *Chlorotetraëdron bitridens* (Beck) Komárek & Kovácik | 0.3 |
| *Cladophora* sp. | 0.6 |
| *Closterium acerosum* Ehrenberg ex Ralfs | 5.8 |
| *Closterium acutum* Brébisson | 1.0 |
| *Closterium acutum* var. *variabile* (Lemmermann) Willi Krieger | 0.6 |
| *Closterium ehrenbergii* Meneghini ex Ralfs | 8.9 |
| *Closterium incurvum* Brébisson | 7.5 |
| *Closterium kuetzingii* Brébisson | 3.0 |
| *Closterium moniliferum* Ehrenberg ex Ralfs | 14.4 |
| *Closterium* sp. | 5.3 |
| *Closterium strigosum* Brébisson | 6.4 |
| *Closterium tumidulum* F.Gay | 4.5 |
| *Coelastrum microporum* Nägeli | 0.8 |
| *Coenococcus planctonicus* Korshikov | 0.3 |
| *Cosmarium dentiferum* Corda ex Nordstedt | 3.6 |
| *Cosmarium granatum* Brébisson ex Ralfs | 3.0 |
| *Cosmarium laeve* Rabenhorst | 0.6 |
| *Cosmarium rectangulare* Grunow | 0.6 |
| *Cosmarium* sp. 1 | 6.4 |
| *Cosmarium* sp. 2 | 3.0 |
| *Cosmarium* sp. 3 | 1.0 |
| *Desmatractum indutum* (Geitler) Pascher | 0.3 |
| *Desmodesmus communis* (E. Hegewald) E. Hegewald | 4.5 |
| *Desmodesmus intermedius* (Chodat) E. Hegewald | 0.6 |
| *Desmodesmus opoliensis* (P. G. Richter) E. Hegewald | 1.0 |
| *Elakatothrix gelatinosa* Wille | 1.4 |
| *Eudorina elegans* Ehrenberg | 1.7 |
| *Follicularia paradoxalis* V. V. Miller | 0.3 |
| *Gonium* sp. | 0.6 |
| *Hariotina reticulata* P. A. Dangeard | 0.8 |
| *Hyaloraphidium contortum* Pascher & Korshikov | 2.0 |
| *Lemmermannia tetrapedia* (Kirchner) Lemmermann | 3.3 |
| *Messastrum gracile* (Reinsch) T. S. Garcia | 0.3 |
| *Microspora* sp. | 0.3 |
| *Monoraphidium contortum* (Thuret) Komárková-Legnerová | 0.8 |
| *Monoraphidium griffithii* (Berkeley) Komárková-Legnerová | 18 |
| *Mougeotia* sp. | 1.7 |
| *Mucidosphaerium pulchellum* (H.C.Wood) C.Bock Proschold & Krienitz | 1.0 |
| *Oedogonium* sp. | 1.9 |
| *Oocystis lacustris* Chodat | 0.3 |
| *Oocystis parva* West & G. S. West | 1.4 |
| *Oocystis* sp. | 2.0 |
| *Pandorina morum* (O. F. Müller) Bory | 6.7 |
| *Pseudoschroederia robusta (Korshikov) E. Hegewald & E. Schnepf* | 2.0 |
| *Pteromonas aculeata* Lemmermann | 6.9 |
| *Raphidocelis danubiana* (Hindák) Marvan Komárek & Comas | 2.8 |
| *Raphidonema sempervirens* Chodat | 0.6 |
| *Scenedesmus ecornis* (Ehrenberg) Chodat | 3.6 |
| *Scenedesmus obtusus* Meyen | 0.6 |
| *Scenedesmus* sp. | 1.4 |
| *Schroederia planctonica* (Skuja) Philipose | 7.8 |
| *Schroederia setigera* (Schröder) Lemmermann | 7.8 |
| *Selenastrum bibraianum* Reinsch | 0.6 |
| *Sphaerocystis planctonica* (Korshikov) Bourrelly | 6.7 |
| *Spirogyra* sp. | 10.0 |
| *Staurastrum bieneanum* Rabenhorst | 0.3 |
| *Staurastrum margaritaceum* Meneghini ex Ralfs | 3.9 |
| *Staurastrum* sp. | 1.7 |
| *Staurodesmus* sp. | 1.4 |
| *Tetradesmus dimorphus* (Turpin) M. J. Wynne | 3.3 |
| *Tetradesmus lagerheimii* M. J. Wynne & Guiry | 1.4 |
| *Tetradesmus obliquus* (Turpin) M. J. Wynne | 0.6 |
| *Tetraëdron caudatum* (Corda) Hansgirg | 0.3 |
| *Tetraëdron minimum* (A. Braun) Hansgirg | 1.9 |
| *Tetraёdron triangulare* Korshikov | 0.6 |
| *Tetrastrum glabrum* (Y. V. Roll) Ahlstrom & Tiffany | 0.6 |
| *Ulothrix zonata* (F. Weber & Mohr) Kützing | 0.6 |
| *Ulothrix* sp. | 5.0 |
| *Uronema confervicola* Lagerheim | 1.0 |
| *Uronema* sp. | 8.0 |
| *Volvox aureus* Ehrenberg | 7.8 |
| coccal chlorophytes | 0.3 |
| **Euglenoids** |  |
| *Astasia* sp. | 3.0 |
| *Colacium mucronatum* Bourrelly & Chadefaud | 0.3 |
| *Colacium vesiculosum* f. *natans* (Lemmermann) Woronichin & Popova | 11.0 |
| *Colacium* sp. | 10.0 |
| *Cryptoglena skujae* Marin & Melkonian | 0.3 |
| *Discoplastis angusta* (C. Bernard) Zakryś & Łukomska | 6.7 |
| *Euglena agilis* H. J. Carter | 2.0 |
| *Euglenaria caudata* (E. F. W. Hübner) Karnkowska-Ishikawa & E. W. Linton | 25.0 |
| *Euglena clara* Skuja | 6.4 |
| *Euglenaria clavata* (Skuja) Karnkowska & E. W. Linton | 19.4 |
| *Euglena deses* (O. F. Müller) Ehrenberg | 21.0 |
| *Euglena ehrenbergii* G .A. Klebs | 3.0 |
| *Euglena gymnodinioides* Zakryś | 1.0 |
| *Euglena hemichromata* Skuja | 11.4 |
| *Euglena ignobilis* L. P. Johnson | 0.3 |
| *Euglena multiformis* J. Schiller | 0.3 |
| *Euglena mutabilis* F. Schmitz | 0.6 |
| *Euglenaformis proxima* (P. A. Dangeard) M. S. Bennett & Triemer | 21.9 |
| *Euglena rostrata* Ehrenberg | 0.3 |
| *Euglena spirogyra* var. *marchica* Lemmermann | 0.6 |
| *Euglena texta* (Dujardin) Hübner | 37.5 |
| *Euglena truncata* L. B. Walton | 0.3 |
| *Euglena viridis* (O. F. Müller) Ehrenberg | 10.0 |
| *Euglena* sp. 1 | 14.5 |
| *Euglena* sp. 2 | 9.4 |
| *Lepocinclis acicularis* Francé | 0.3 |
| *Lepocinclis acus* (O. F. Müller) B. Marin & Melkonian | 10.0 |
| *Lepocinclis cyclidiopsis* M. S. Bennett & Triemer | 0.6 |
| *Lepocinclis fusiformis* (H. J. Carter) Lemmermann | 12.5 |
| *Lepocinclis globulus* Perty | 0.3 |
| *Lepocinclis hispidula (Eichwald) Daday* | 0.3 |
| *Lepocinclis longissima* (Deflandre) Zakryś & Chaber | 1.0 |
| *Lepocinclis ovum (Ehrenberg) Lemmermann* | 14.7 |
| *Lepocinclis oxyuris* (Schmarda) B. Marin & Melkonian | 10.6 |
| *Lepocinclis spirogyroides* B. Marin & Melkonian | 14.2 |
| *Lepocinclis steinii* (Lemmermann) Lemmermann | 19.7 |
| *Lepocinclis tripteris (Dujardin)* B. Marin & M. Melkonian | 30.55 |
| *Lepocinclis tschernovii* Popowa | 0.3 |
| *Lepocinclis* sp. 1 | 7.8 |
| *Lepocinclis* sp. 2 | 0.8 |
| *Menoidium tortuosum* (A. Stokes) Lemmermann | 0.3 |
| *Monomorphina pyrum (Ehrenberg) Mereschkowsky* | 1.9 |
| *Monomorphina* sp. 1 | 2.5 |
| *Phacus acuminatus* A.Stokes | 22.8 |
| *Phacus alatus* G. A. Klebs | 25.0 |
| *Phacus arnoldii* Svirenko | 0.6 |
| *Phacus caudatus* Hübner | 18.9 |
| *Phacus clavatus* P. A. Dangeard | 0.6 |
| *Phacus corculum* Pochmann | 0.3 |
| *Phacus curvicauda* Svirenko | 3.0 |
| *Phacus elegans* Pochmann | 2.5 |
| *Phacus limnophilus* (Lemmermann) E. W. Linton & Karnkowska | 5.0 |
| *Phacus longicauda* (Ehrenberg) Dujardin | 21.7 |
| *Phacus monilatus* var. *suecicus* Lemmermann | 3.3 |
| *Phacus onyx* Pochmann | 0.3 |
| *Phacus orbicularis* Hübner | 19.2 |
| *Phacus parvulus* G. A. Klebs | 1.0 |
| *Phacus pusillus* Lemmermann | 0.3 |
| *Phacus smulkowskianus* (Zakryś) W.-H. Kusber | 0.6 |
| *Phacus* sp. 1 | 0.6 |
| *Phacus* sp. 2 | 5.55 |
| *Phacus* sp. 3 | 3.3 |
| *Phacus* sp. 4 | 0.8 |
| *Strombomonas acuminata* (Schmarda) Deflandre | 0.3 |
| *Strombomonas* sp. | 1.4 |
| *Trachelomonas abrupta* Svirenko | 2.0 |
| *Trachelomonas abrupta* var. *minor* Deflandre | 0.3 |
| *Trachelomonas armata* (Ehrenberg) F. Stein | 15.6 |
| *Trachelomonas armata* var. *setosa* Dreżepolski | 0.3 |
| *Trachelomonas australica var. granulata* (Playfair) Deflandre | 10.0 |
| *Trachelomonas caudata* (Ehrenberg) F. Stein | 15.3 |
| *Trachelomonas cylindrica* Ehrenberg | 8.3 |
| *Trachelomonas dubia*Svirenko | 0.6 |
| *Trachelomonas duplex* (Deflandre) Couté & Tell | 0.6 |
| *Trachelomonas dybowskii* Dreżepolski | 0.3 |
| *Trachelomonas globularis* (Averintsev) Lemmermann | 3.3 |
| *Trachelomonas globularis* f. *crenulatocollis* (Szabados) T. G. Popova | 1.0 |
| *Trachelomonas hexangulata* Svirenko | 0.3 |
| *Trachelomonas hispida* (Perty) F. Stein | 48.0 |
| *Trachelomonas hispida* var. *coronata* Lemmermann | 1.9 |
| *Trachelomonas hispida* var. *crenulatocollis* (Maskell) Lemmermann | 5.8 |
| *Trachelomonas hispida* var. *volicensis* Dreżepolski | 0.3 |
| *Trachelomonas intermedia* P. A. Dangeard | 42.2 |
| *Trachelomonas irregularis* Svirenko | 0.6 |
| *Trachelomonas klebsii* Deflandre | 0.8 |
| *Trachelomonas lacustris* Dreżepolski | 0.6 |
| *Trachelomonas manginii* Deflandre | 9.4 |
| *Trachelomonas oblonga* Lemmermann | 22.5 |
| *Trachelomonas oblonga* var. *pulcherrima* (Playfair) T. G. Popova | 13.6 |
| *Trachelomonas obtusa* T. C. Palmer | 0.3 |
| *Trachelomonas planctonica* Svirenko | 0.3 |
| *Trachelomonas pseudobulla* Svirenko | 5.3 |
| *Trachelomonas pusilla* Playfair | 18.0 |
| *Trachelomonas robusta* Svirenko | 0.3 |
| *Trachelomonas rugulosa* F. Stein | 4.5 |
| *Trachelomonas similis* A. Stokes | 2.5 |
| *Trachelomonas spinulosa* (Skvortsov) Deflandre | 1.9 |
| *Trachelomonas stokesii* Dreżepolski | 0.3 |
| *Trachelomonas subverrucosa* Deflandre | 0.6 |
| *Trachelomonas sydneyensis* Playfair | 2.5 |
| *Trachelomonas verrucosa* A. Stokes | 4.5 |
| *Trachelomonas verrucosa* var*. spirogyra* (Balech) Huber-Pestalozzi | 0.3 |
| *Trachelomonas volvocina* (Ehrenberg) Ehrenberg | 19.7 |
| *Trachelomonas volvocina* var. *derephora* W. Conrad | 28.3 |
| *Trachelomonas volvocinopsis* Svirenko | 40.3 |
| *Trachelomonas woycickii* Koczwara | 5.8 |
| *Trachelomonas* sp. 1 | 3.9 |
| *Trachelomonas* sp. 2 | 3.3 |
| *Trachelomonas* sp. 3 | 1.9 |
| *Trachelomonas* sp. 4 | 0.3 |
| *Trachelomonas* sp. 5 | 9.2 |
| *Trachelomonas* sp. 6 | 27.2 |
| *Trachelomonas* sp. 7 | 3.9 |
| **Diatoms** |  |
| *Achnanthes* sp. | 11.7 |
| *Achnanthidium affine* (Grunow) Czarnecki | 1.7 |
| *Achnanthidium lineare* W. Smith | 0.3 |
| *Achnanthidium minutissimum (*Kützing) Czarnecki | 3.0 |
| *Caloneis amphisbaena* (Bory) Cleve | 0.3 |
| *Caloneis molaris* (Grunow) Krammer | 0.3 |
| *Caloneis silicula* (Ehrenberg) Cleve | 1.7 |
| *Chamaepinnularia begeri* (Krasske) Lange-Bertalot | 0.3 |
| *Cocconeis placentula* Ehrenberg | 0.8 |
| *Craticula cuspidata* (Kutzing) D. G. Mann | 8.6 |
| *Cyclotella* sp. | 0.8 |
| *Cymbella ventricosa* Kützing | 0.3 |
| *Denticula* sp. | 0.3 |
| *Epithemia adnata* (Kützing) Brébisson | 2.0 |
| *Epithemia argus* (Ehrenberg) Kützing | 0.3 |
| *Epithemia gibba* (Ehrenberg) Kützing | 1.0 |
| *Epithemia turgida* (Ehrenberg) Kützing | 0.3 |
| *Eucocconeis flexella* (Kützing) F. Meister | 1.4 |
| *Eunotia arcubus* var. *bidens* (Grunow) Lange-Bertalot | 0.3 |
| *Eunotia bilunaris* (Ehrenberg) Schaarschmidt | 50.0 |
| *Eunotia curtagrunowii* Nörpel-Schempp & Lange-Bertalot | 0.3 |
| *Eunotia exigua* (Brébisson ex Kützing) Rabenhorst | 1.4 |
| *Eunotia inflata* (Grunow) Norpel-Schempp & Lange-Bertalot | 0.6 |
| *Eunotia lunaris* var. *capitata* (Grunow) Schönfeldt | 0.3 |
| *Eunotia pectinalis* (Kützing) Rabenhorst | 0.3 |
| *Eunotia praerupta* Ehrenberg | 4.5 |
| *Eunotia* sp. 1 | 8.0 |
| *Eunotia* sp. 2 | 1.0 |
| *Eunotia* sp. 3 | 0.6 |
| *Fragilaria capucina* Desmazières | 0.8 |
| *Fragilaria intermedia* (Grunow) Grunow | 1.0 |
| *Fragilaria* sp. | 0.8 |
| *Fragilariforma virescens* (Ralfs) D. M. Williams & Round | 2.0 |
| *Gogorevia exilis* (Kützing) Kulikovskiy & Kociolek | 2.5 |
| *Gomphonema acuminatum* Ehrenberg | 0.3 |
| *Gomphonema augur* Ehrenberg | 16 |
| *Gomphonema intricatum* Kützing | 1.0 |
| *Gomphonella olivacea* (Hornemann) Rabenhorst | 0.3 |
| *Gomphonema parvulum* (Kützing) Kützing | 0.3 |
| *Hantzschia amphioxys* (Ehrenberg) Grunow | 24.7 |
| *Hantzschia amphioxys* f. *capitata* O. Müller | 11.7 |
| *Hantzschia amphioxys* var. *minor* H. Peragallo | 3.3 |
| *Karayevia laterostrata* (Hustedt) Bukhtiyarova | 0.3 |
| *Kobayasiella subtilissima* (Cleve) Lange-Bertalot | 0.8 |
| *Meridion circulare* (Greville) C. Agardh | 1.0 |
| *Navicula cincta* Pantocsek | 11.0 |
| *Navicula cryptocephala* Kützing | 1.4 |
| *Navicula disjuncta* Hustedt | 0.3 |
| *Navicula exigua* W. Gregory | 1.7 |
| *Navicula gregaria* Donkin | 0.8 |
| *Navicula minima* Grunow | 5.8 |
| *Navicula radiosa* Kützing | 0.3 |
| *Navicula rhynchocephala* Kützing | 0.3 |
| *Navicula tripunctata* (O. F. Müller) Bory | 0.8 |
| *Navicula* sp. 1 | 25.0 |
| *Navicula* sp. 2 | 20.0 |
| *Navicula* sp. 3 | 4.0 |
| *Neidium productum* (W. Smith) Cleve | 0.3 |
| *Nitzschia acicularis* (Kützing) W. Smith | 0.8 |
| *Nitzschia fonticola* (Grunow) Grunow | 0.3 |
| *Nitzschia palea* (Kützing) W. Smith | 65.3 |
| *Nitzschia recta*Hantzsch ex Rabenhorst | 0.8 |
| *Nitzschia sigmoidea* (Nitzsch) W. Smith | 0.6 |
| *Nitzschia thermalis* (Ehrenberg) Auerswald | 2.0 |
| *Nitzschia thermalis* var. *minor* Hilse | 14.2 |
| *Nitzschia* sp. | 1.4 |
| *Paraplaconeis placentula* (Ehrenberg) Kulikovskiy & Lange-Bertalot | 0.3 |
| *Pinnularia abaujensis* var. *subundulata* (Ant. Mayer) R. M. Patrick | 2.0 |
| *Pinnularia biceps* f. *minutissima* (Hustedt) A. Cleve | 10.6 |
| *Pinnularia borealis* Ehrenberg | 6.4 |
| *Pinnularia infirma* Krammer | 0.3 |
| *Pinnularia interrupta* W. Smith | 0.6 |
| *Pinnularia major* (Kützing) Rabenhorst | 9.4 |
| *Pinnularia major* var. *lacustris* F. Meister | 2.0 |
| *Pinnularia mesolepta* (Ehrenberg) W. Smith | 21.7 |
| *Pinnularia microstauron* (Ehrenberg) Cleve | 5.8 |
| *Pinnularia nobilis* (Ehrenberg) Ehrenberg | 0.3 |
| *Pinnularia schoenfelderi* Krammer | 0.3 |
| *Pinnularia subcapitata*W. Gregory | 1.4 |
| *Pinnularia viridis* (Nitzsch) Ehrenberg | 30.0 |
| *Pinnularia* sp. | 6.7 |
| *Placoneis dicephala* (Ehrenberg) Mereschkowsky | 0.8 |
| *Planothidium lanceolatum* (Brébisson ex Kützing) Lange-Bertalot | 0.3 |
| *Rhoicosphenia abbreviata* (C. Agardh) Lange-Bertalot | 0.3 |
| *Sellaphora parapupula* Lange-Bertalot | 0.3 |
| *Sellaphora pupula* f. *capitata* (Skvortzov & K. I. Meyer) Poulin | 0.3 |
| *Stauroneis anceps* Ehrenberg | 26.7 |
| *Stauroneis dilatata* Ehrenberg | 0.3 |
| *Stauroneis phoenicenteron* (Nitzsch) Ehrenberg | 28.0 |
| *Stauroneis phoenicenteron* f. *gracilis* Hustedt | 2.5 |
| *Stauroneis* sp. 1 | 0.3 |
| *Stauroneis* sp. 2 | 3.6 |
| *Staurosira construens* Ehrenberg | 0.8 |
| *Tabellaria* sp. | 0.3 |
| *Ulnaria acus* (Kützing) Aboal | 2.0 |
| *Ulnaria ulna* (Nitzsch) Compère | 4.0 |
| **Cryptophytes** |  |
| *Chilomonas insignis* (Skuja) Javornický | 1.9 |
| *Chilomonas* sp. | 0.3 |
| *Chroomonas minima* Czosnowski | 0.6 |
| *Chroomonas* sp. 1 | 1.7 |
| *Chroomonas* sp. 2 | 5.0 |
| *Chroomonas* sp. 3 | 0.6 |
| *Cryptomonas caudata* Massart | 0.6 |
| *Cryptomonas commutata* (Pascher) Hoef-Emden | 4.0 |
| *Cryptomonas curvata* Ehrenberg | 19.0 |
| *Cryptomonas erosa* Ehrenberg | 60.0 |
| *Cryptomonas gracilis* Skuja | 1.9 |
| *Cryptomonas marssonii* Skuja | 57.5 |
| *Cryptomonas obovata* Skuja | 0.3 |
| *Cryptomonas ovata* Ehrenberg | 24.4 |
| *Cryptomonas ovata* var. *splendida* (Czosnowski) Javornický | 0.6 |
| *Cryptomonas phaseolus* Skuja | 0.3 |
| *Cryptomonas platyuris* Skuja | 0.3 |
| *Cryptomonas pyrenoidifera* Geitler | 1.0 |
| *Cryptomonas similis* Hollande | 0.3 |
| *Cryptomonas tenuis* Pascher | 1.4 |
| *Cryptomonas woloszynskae* J. Czosnowski | 1.7 |
| *Cryptomonas* sp. 1 | 4.7 |
| *Cryptomonas* sp. 2 | 0.3 |
| *Cryptomonas* sp. 3 | 0.6 |
| *Cyanomonas acuta* (J. Schiller) J. Schiller | 0.8 |
| *Komma caudata* (L. Geitler) D. R. A. Hill | 1.7 |
| *Rhodomonas pusilla* (Bachmann) Javornický | 12.0 |
| *Rhodomonas tenuis* Skuja | 13.0 |
| *Rhodomonas* sp. | 1.9 |
| **Dinophlagellates** |  |
| *Gymnodinium albulum* Lindemann | 0.3 |
| *Gymnodinium discoidale* T. M. Harris | 0.3 |
| *Gymnodinium simile* Skuja | 0.3 |
| *Gymnodinium uberrimum* (G. J. Allman) Kofoid & Swezy | 1.4 |
| *Gymnodinium* sp. 1 | 13.0 |
| *Gymnodinium* sp. 2 | 1.0 |
| *Gymnodinium* sp. 3 | 0.3 |
| *Jadwigia hiemalis* (Wołoszyńska) Moestrup | 0.3 |
| *Nusuttodinium acidotum* (Nygaard) Y. Takano & T. Horiguchi | 3.0 |
| *Peridinium cinctum* (O. F. Müller) Ehrenberg | 1.7 |
| *Peridinium umbonatum* Karsten | 0.3 |
| *Peridinium* sp. | 2.0 |
| *Woloszynskia* sp. | 9.7 |
| cysts | 5.0 |
| **Chrysophyceae** |  |
| *Chrysochromulina parva* Lackey | 1.7 |
| *Dinobryon divergens* O. E. Imhof | 0.8 |
| *Dinobryon sociale* (Ehrenberg) Ehrenberg | 6.0 |
| *Ochromonas* sp. | 10.0 |
| *Uroglena* sp. | 0.3 |
| **Xanthophytes** |  |
| *Ophiocytium arbuscula* (A.Braun ex Kützing) Sande Lacoste & Suringar | 0.3 |
| *Ophiocytium cochleare* (Eichwald) A. Braun | 6.4 |
| *Pseudostaurastrum enorme* (Ralfs) Chodat | 0.3 |
| *Tribonema minus* (Wille) Hazen | 0.6 |
| *Tribonema* sp. 1 | 11.9 |
| *Tribonema* sp. 2 | 1.9 |
