# Supplementary material for: Shifts in phytoplankton communities in response to water parameters and large branchiopod filter feeders in kettle hole ponds of farmland landscape
Source: Sci Rep. 2025 May 21;15:17623. doi: 10.1038/s41598-025-01060-9 (PMC12095657; doi:10.1038/s41598-025-01060-9)
Supplement: Supplementary file 2 — Supplementary Material 2 [file 41598_2025_1060_MOESM2_ESM.docx]

**Supplementary Material S2.** Temporal changes in phytoplankton abundance in the investigated kettle hole ponds.

1. Changes in phytoplankton abundance in BRE pond.
2. Changes in phytoplankton abundance in BRW pond.
3. Changes in phytoplankton abundance in DRD pond.
4. Changes in phytoplankton abundance in DRM pond.
5. Changes in phytoplankton abundance in IRS pond.
6. Changes in phytoplankton abundance in STR pond.
7. Changes in phytoplankton abundance in TPG pond.
8. Changes in phytoplankton abundance in TPS pond.
9. Changes in phytoplankton abundance in TRI pond.
