# Supplementary material for: Shifts in phytoplankton communities in response to water parameters and large branchiopod filter feeders in kettle hole ponds of farmland landscape
Source: Sci Rep. 2025 May 21;15:17623. doi: 10.1038/s41598-025-01060-9 (PMC12095657; doi:10.1038/s41598-025-01060-9)
Supplement: Supplementary file 3 — Supplementary Material 3 [file 41598_2025_1060_MOESM3_ESM.docx]

**Supplementary Material S3.** Temporal changes in the contribution of phytoplankton groups to the total numbers of phytoplankton in the investigated kettle hole ponds, displayed as ratio between the abundance of a group to the total phytoplankton abundance in the sample (Y-axis presents dominance structure or contribution of the groups in %). Blue arrows represent inundation events, x = missing sample.


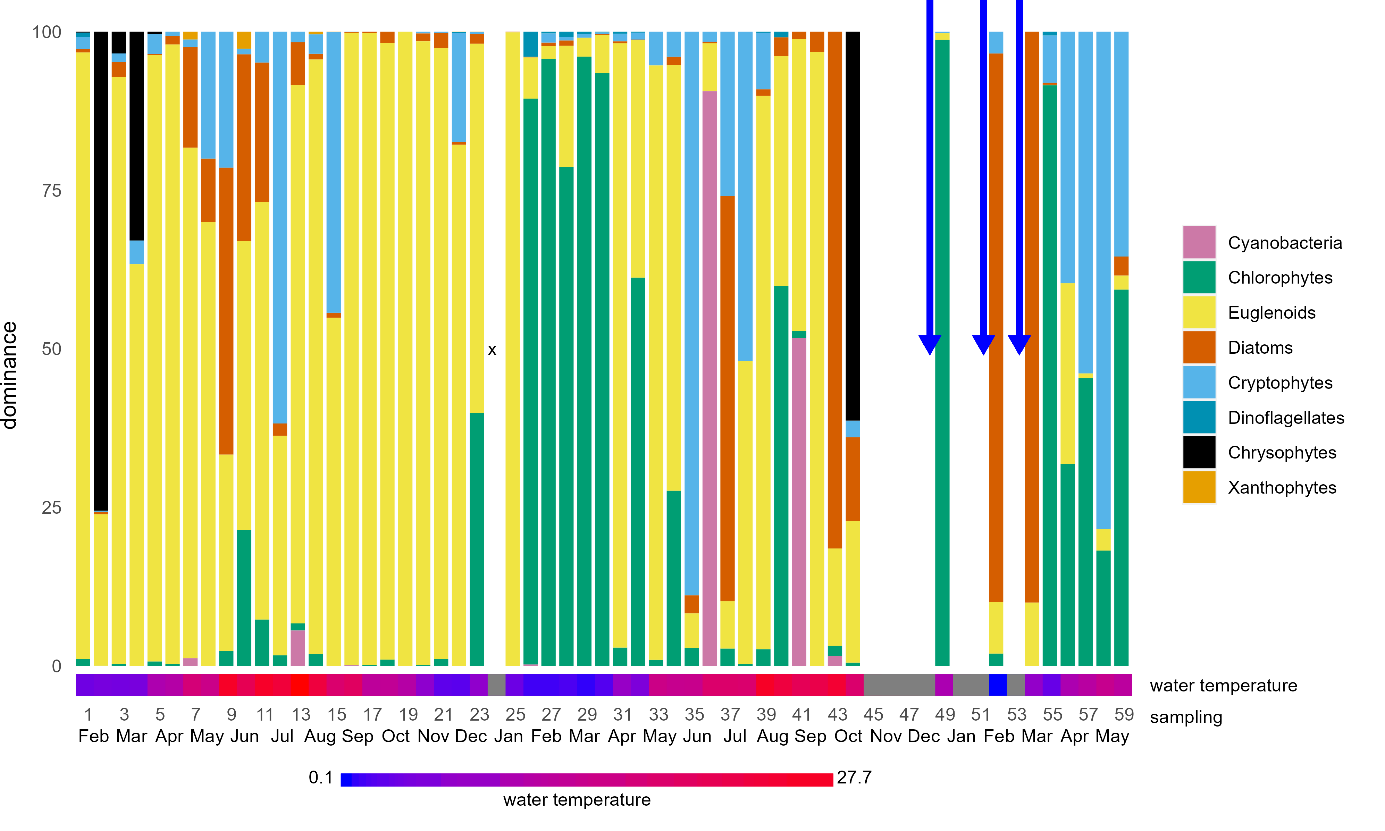


1. Changes in phytoplankton dominance in BRE pond
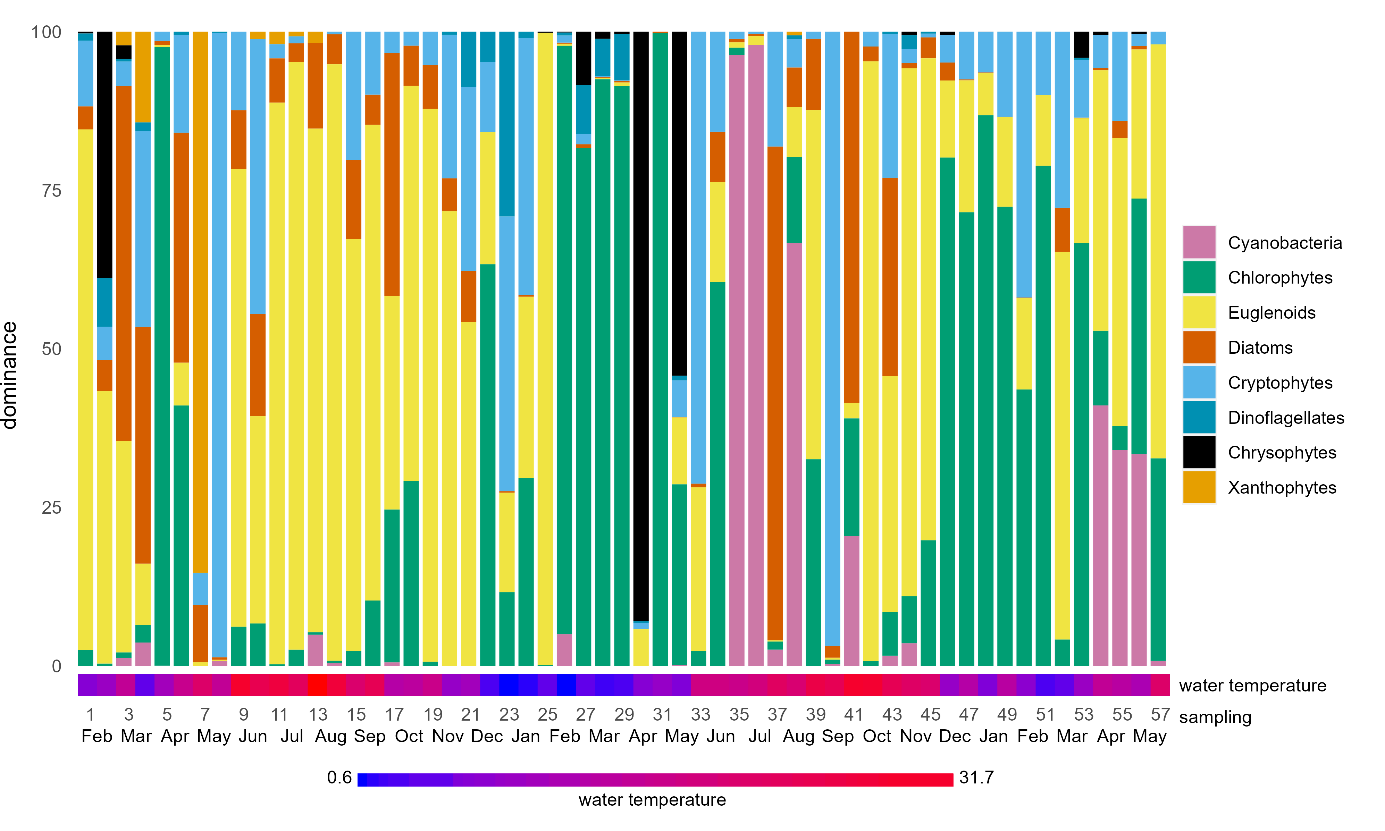

2. Changes in phytoplankton dominance in BRW pond


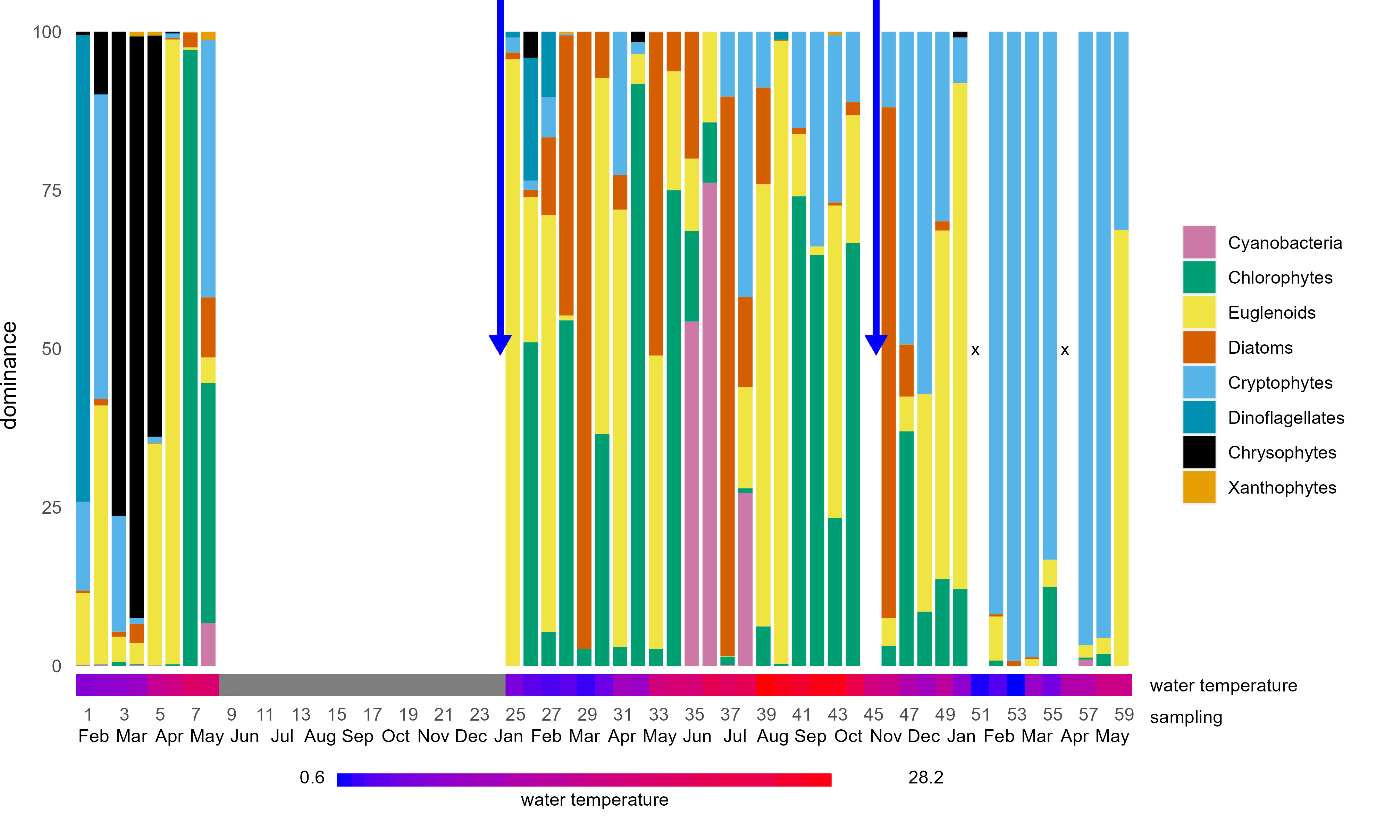


1. Changes in phytoplankton dominance in DRD pond


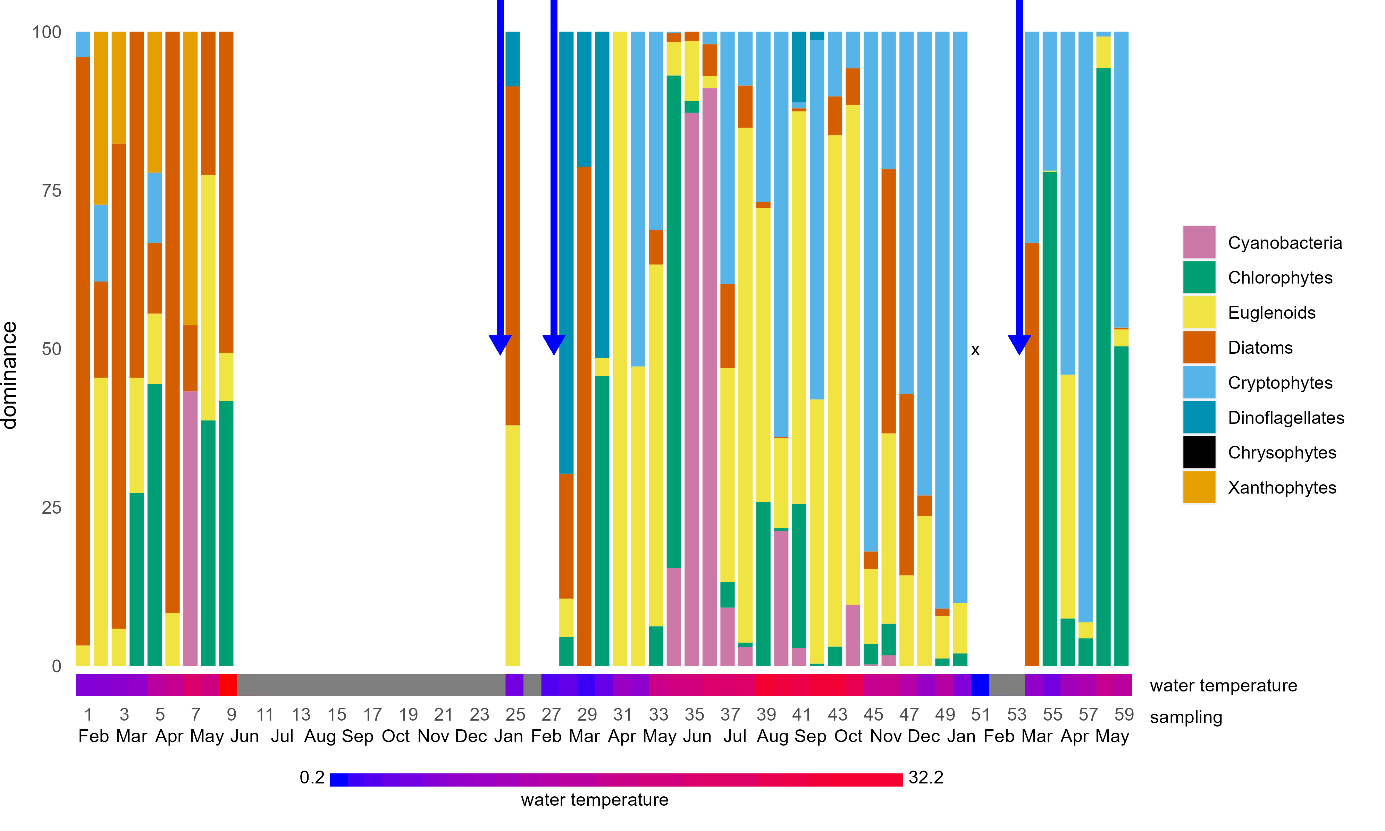


1. Changes in phytoplankton dominance in DRM pond


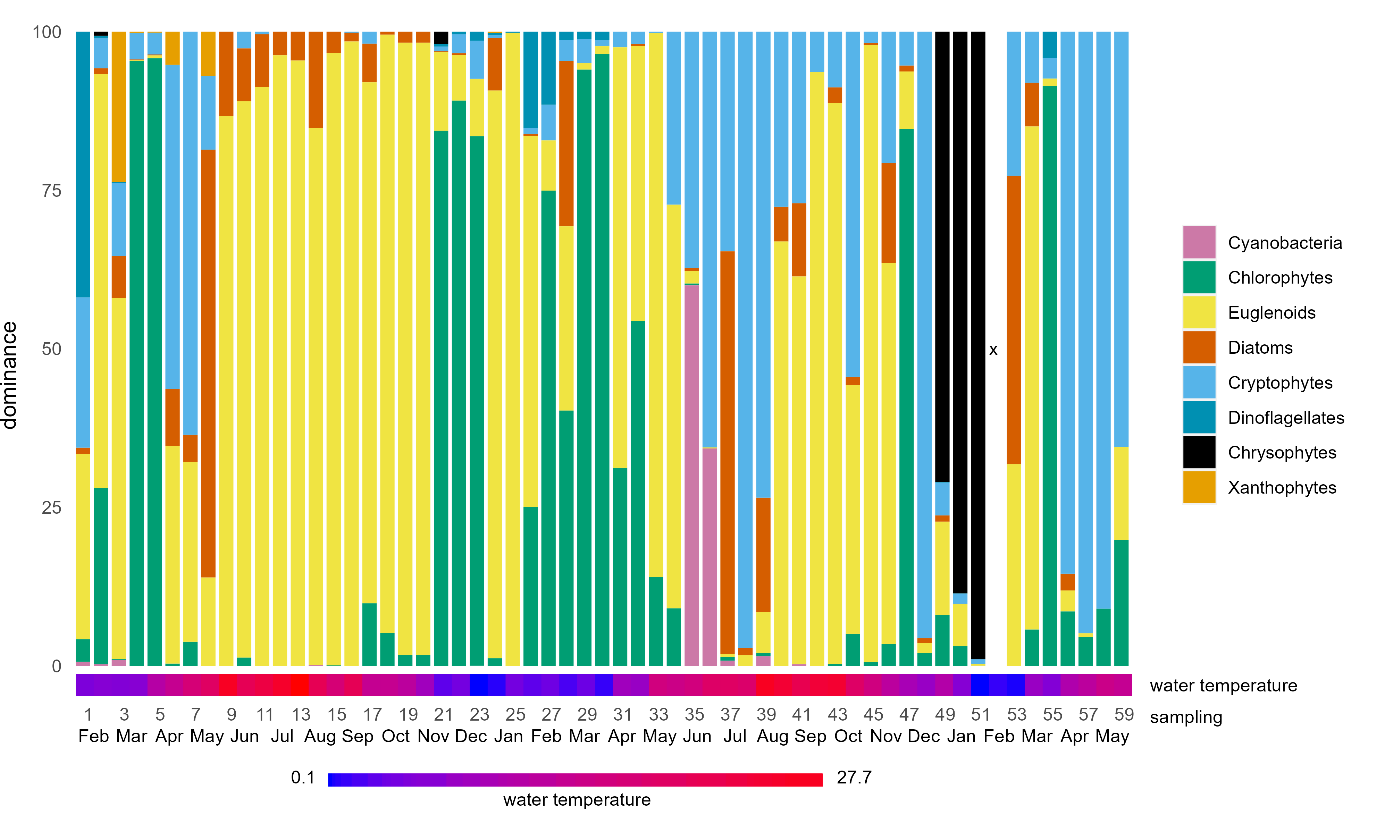


1. Changes in phytoplankton dominance in IRS pond


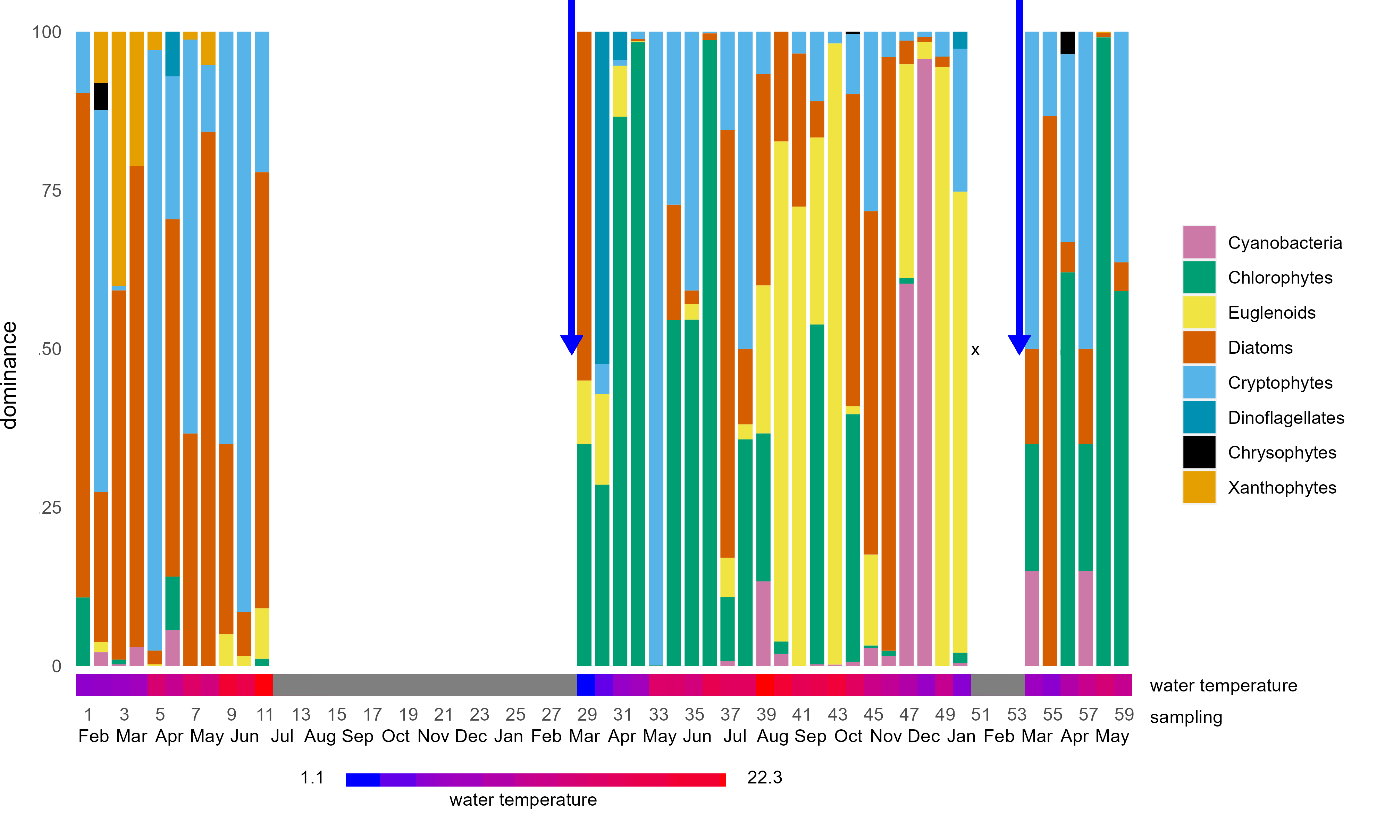


1. Changes in phytoplankton dominance in STR pond


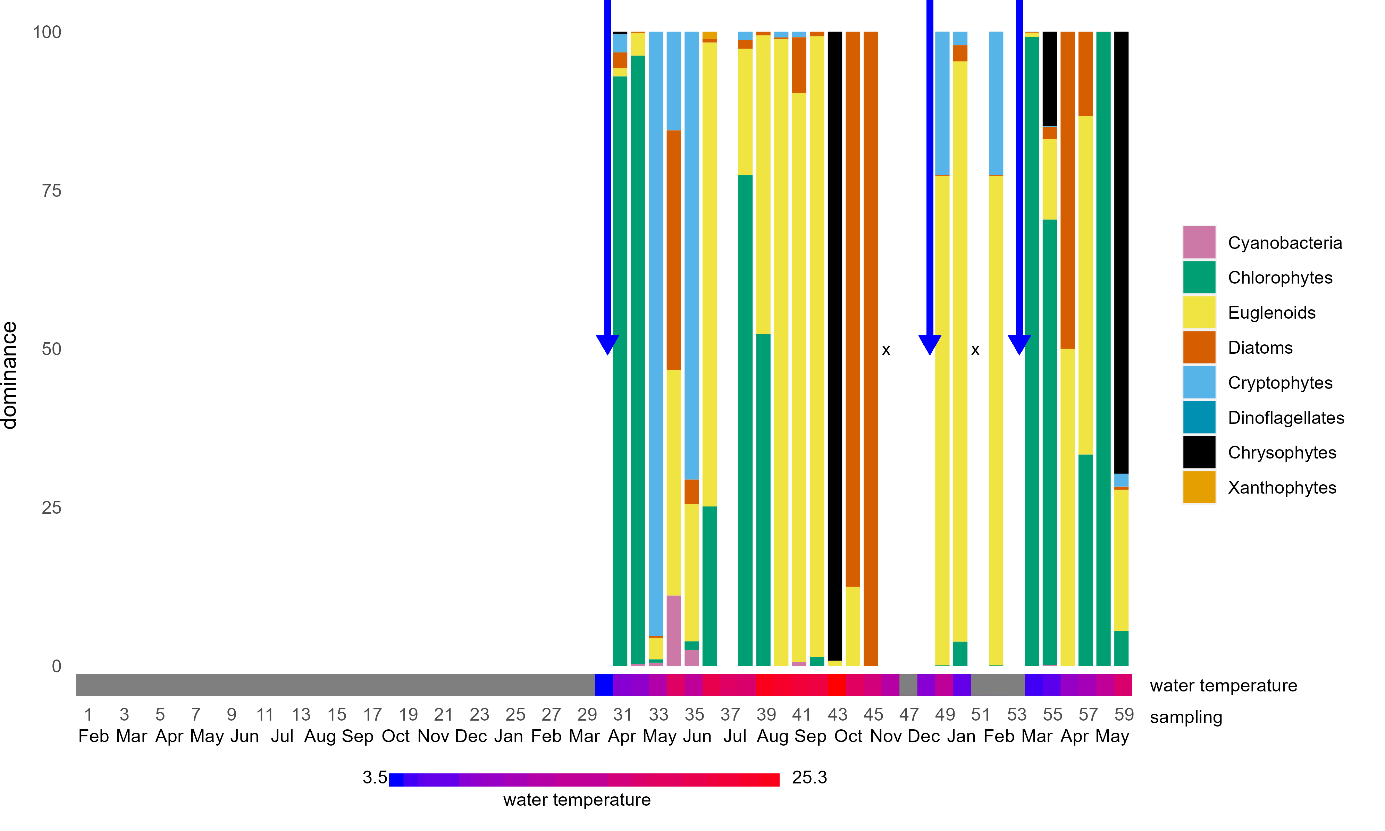


1. Changes in phytoplankton dominance in TPG pond


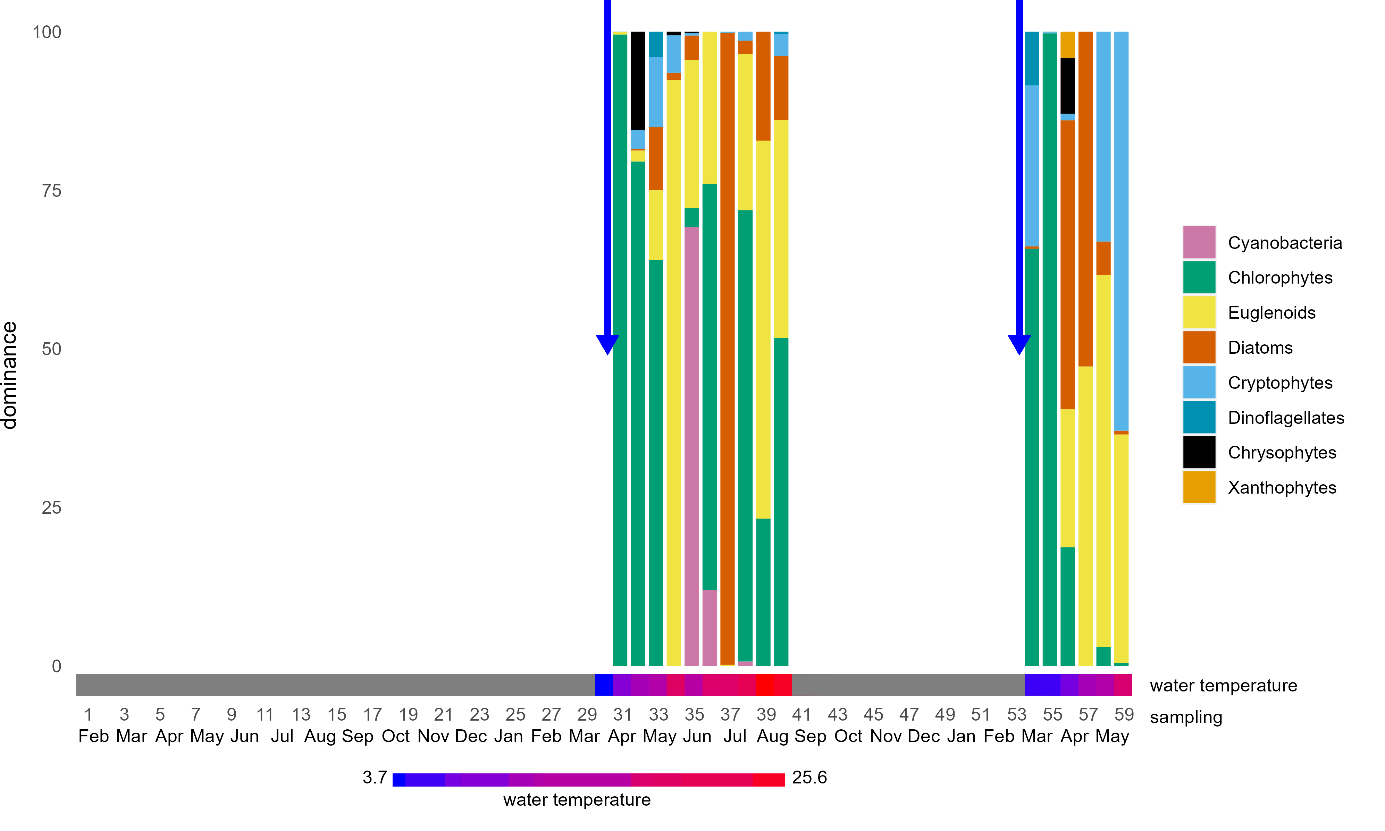


1. Changes in phytoplankton dominance in TPS pond


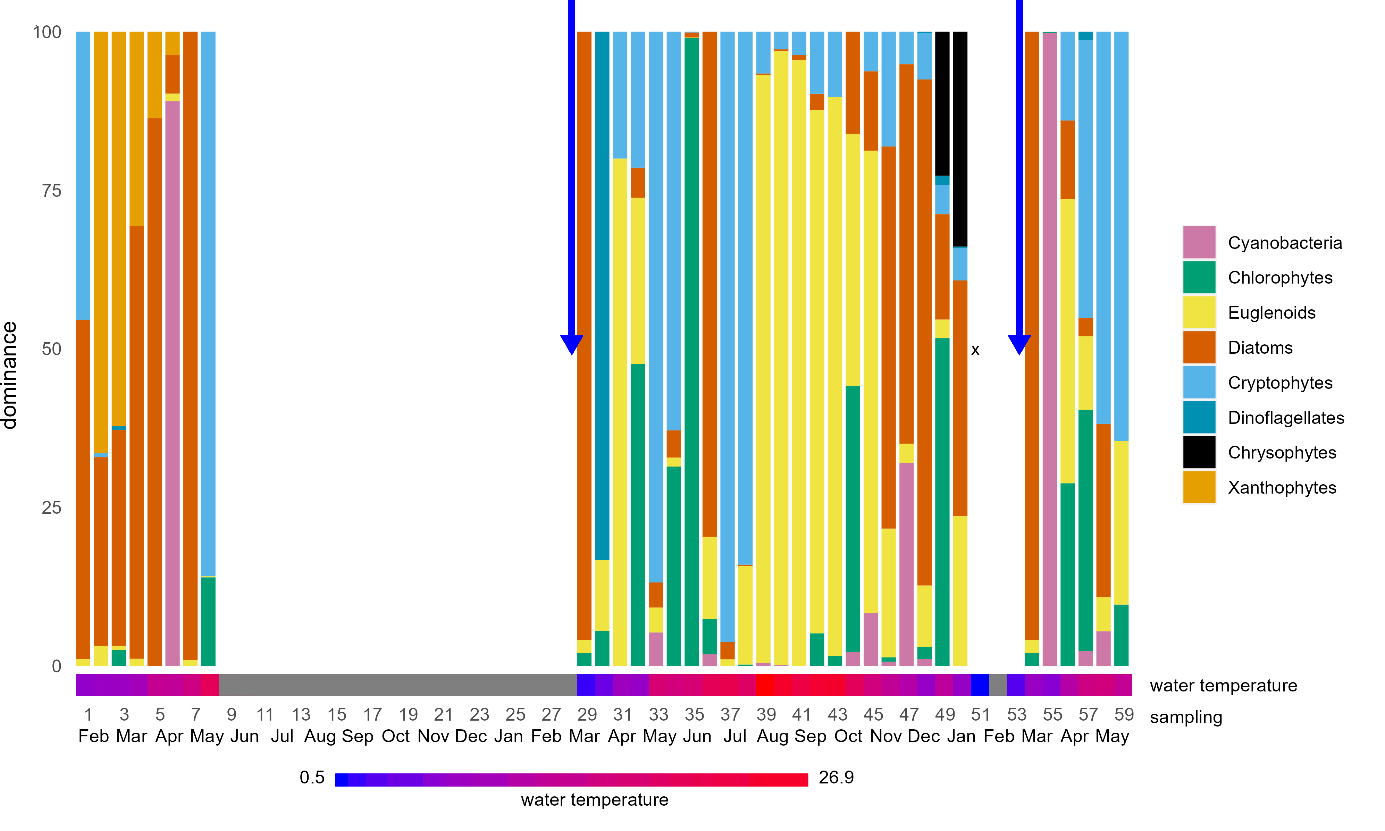


1. Changes in phytoplankton dominance in TRI pond
